# A Hydrophobic Cluster Modulates Long-Range Allostery in the TRMT2A RNA Recognition Motif

**DOI:** 10.1021/acs.jcim.5c02753

**Published:** 2026-05-12

**Authors:** Mohammed Khaled, Lisa Johannknecht, Oscar Palomino-Hernandez

**Affiliations:** † Department of Chemistry, 9182 Johannes Gutenberg University Mainz, Duesbergweg 10-14, Mainz D-55128, Germany; ‡ Institute for Quantitative and Computational Biosciences (IQCB), Johannes Gutenberg University Mainz, Johannes-von-Müller-Weg 6, Mainz D-55128, Germany

## Abstract

TRMT2A has emerged as a disease-modifying target in polyglutamine (PolyQ) models, yet the conformational preferences and allostery of its RNA recognition motif (RRM) remain poorly resolved. Here, we combine extensive atomistic molecular dynamics with Markov state modeling (MSM), transition path theory, and structure-based pocket analysis to map the conformational landscape of the human TRMT2A RRM. We resolve six metastable states and show that a hydrophobic cluster centered on F92–W134–L133 modulates their interconversion. We further identify residues that contribute to RNA strand recognition and reveal state-specific cryptic pockets consistent with the reported binding sites of TRMT2A RRM small-molecule inhibitors. Together, these results support a hinge–gate model in which a soft, defect-enabled α2 segment and a loop 5 hydrophobic cluster coordinate long-range communication between the ribonucleoprotein (RNP) face and the opposite side, yielding testable mutational predictions and state-specific opportunities for allosteric control of TRMT2A in polyQ disease contexts.

## Graphical abstract

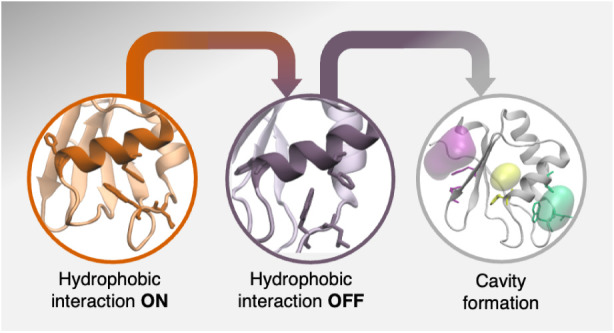

## Introduction

Polyglutamine (polyQ) diseases are a subset of trinucleotide repeat expansion disorders. These expansions produce abnormally long polyQ tracts in the corresponding proteins, which adopt β-sheet–rich conformations and aggregate into insoluble inclusion bodies, hallmarks of neuronal pathology.^1,2^ Mounting evidence indicates that pathways controlling RNA processing and translation can modulate polyQ toxicity, highlighting RNA–protein interactions as promising therapeutic entry points. In this context, *h*TRMT2A—a tRNA m^5^U methyltransferase—has emerged as a potential target for polyQ toxicity.^3^ *h*TRMT2A catalyzes the methylation of U_54_ in the tRNA T-loop to form m^5^U_54_, an RNA modification essential for tRNA stability and translational fidelity.^4,5^ Thus, rather than directly engaging with polyQ sequences or their transcripts, inhibition of *h*TRMT2A mitigates polyQ-associated phenotypes by limiting the production of the expanded polyQ proteins.

The RNA binding process of *h*TRMT2A is primarily mediated by its N-terminal RNA recognition motif (RRM).^4,5^ This RRM adopts the canonical β1−α1−β2−β3−α2−β4 fold, as other RRMs (Figure 1).^6^ This domain contains two highly conserved short sequence motifs known as ribonucleoprotein 1 (RNP1) and ribonucleoprotein 2 (RNP2), on the β3 and β1 motifs, respectively. Aromatic residues within these motifs, such as F106 and F113, engage in π–π stacking with nucleobases and can intercalate between adjacent bases to stabilize binding.^7,8^

**Figure 1:**
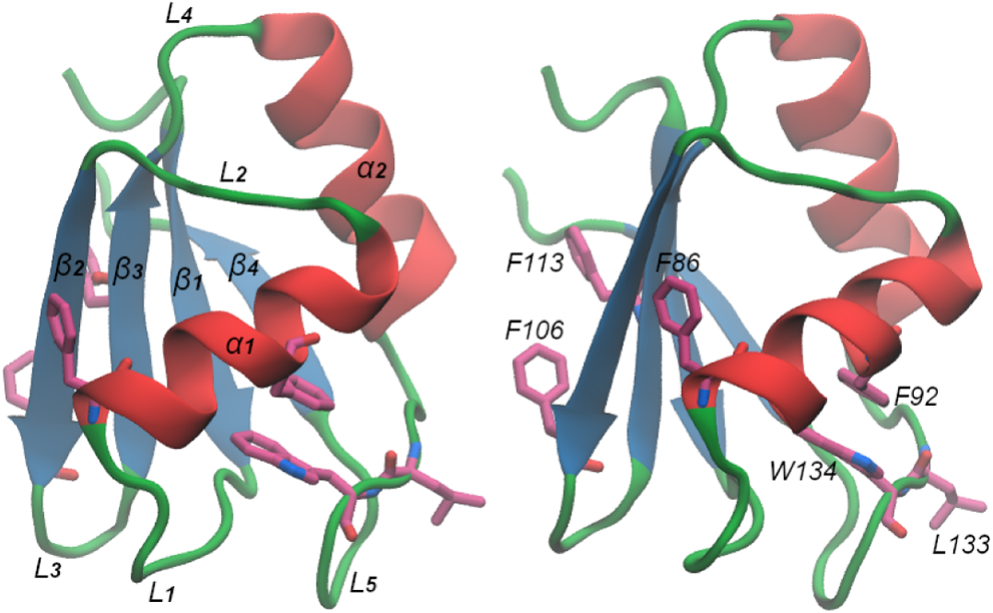
*h*TRMT2A representative structure shown in two views. The protein is displayed in cartoon representation, with secondary-structure elements highlighted as β-strands (blue), α-helices (red), and loops (green). Key residues (F86, F92, F106, F113, L133, and W134) are shown as sticks. In the left panel, secondary-structure elements are labeled, while in the right panel, the same structure is shown from an alternative view, highlighting and labeling the key residues.

X-ray structures of the isolated *h*TRMT2A RRM domain (PDB: 7NTO, 7NTN) revealed the atomistic details of this domain and suggested the existence of a cryptic binding site located between the α1 helix and loop 5, opposite to the RNP1/2 RNA-binding surface. Furthermore, this work also hypothesized an allosteric communication mechanism connecting the RNP on one side and W134 from loop 5 in the opposite cryptic site, despite their considerable spatial separation.^9^

Cryptic pockets of this type are typically transient and sparsely populated in equilibrium ensembles, making them difficult to assess from static structures alone. However, cryptic pockets also show strong potential as therapeutic target sites, offering opportunities to address otherwise *undruggable* proteins.^10,11^ Capturing their formation and coupling requires methods that resolve rare, slow transitions. While classical molecular dynamic simulations (MD simulations) affords atomistic detail, trajectories often under sample millisecond–second events.^12,13^ Markov State Modeling (MSM) addresses this limitation by combining multiple trajectories into a single kinetic model that maps metastable basins and their interconversion rates.^14^

Here, we combine extensive atomistic MD simulations with MSM analysis to delineate the conformational landscape of the *h*TRMT2A RRM. We identified six metastable states, of which two dominate the equilibrium population and serve as kinetic hubs. We also uncover robust allosteric communication between the RNP1/2 face and the opposing surface, mediated by a compact hydrophobic cluster. Together, these results support a mechanistic model in which this internal hydrophobic switch can impact the conformational landscape of the RNP1/2 face, reshaping the RNA-binding surface in a state-dependent manner. By localizing the key residues and state-specific conformations underlying the coupling between transitions among conformational states and the formation of state-specific cryptic pockets, our work provides testable hypotheses for mutational validation and highlights actionable sites for allosteric modulation in the context of polyQ disease biology.

## Materials and Methods

### Simulation Settings of the *h*TRMT2A RRM Motif

To comprehensively sample the conformational landscape of *h*TRMT2A, we employed an iterative simulation and clustering protocol. The process was initiated by selecting 50 distinct conformations from a prior replica-exchange simulation ensemble.^9^ Each of these structures was then used to seed independent 1000 ns MD simulations. Following this, the resulting trajectories were subjected to geometric clustering via their carbon-alpha atoms using GROMOS,^15^ default settings, to identify 50 new representative conformations. This entire procedure—running 50 simulations followed by clustering—was repeated two more times, for a total of three cycles. Each MD simulations trajectory in all three cycles was 1000 ns. The final iteration yielded a refined set of 150 trajectories for subsequent analysis.

For each conformation, the system was prepared as follows: The protonation states of the residues were determined using PROPKA.^16,17^ The system was described using the AMBER ff14SB force field,^18^ at 4 fs time-step through hydrogen mass repartitioning.^19^ The system was solvated in a dodecahedral box with TIP3P water molecules,^20^ and Joung-Cheatham (JC) counterions (Cl^–^ and Na^+^) were added to neutralize the system to a concentration of 0.15 mol/L.^21^ The systems were prepared prior to the production run through energy minimization, 1 ns NVT equilibration, and 1 ns NPT equilibration. Temperature control was managed with the Bussi-Donadio-Parrinello thermostat at 300 K, coupling constant 0.5 ps,^22^ while pressure control was done with the Bernetti-Bussi barostat at 1 atm, coupling constant 5.0 ps.^23^ The SHAKE algorithm was used to constrain bond lengths involving hydrogen atoms.^24^ An 8 Å cutoff was used for vdW and electrostatic interactions, with particle mesh Ewald (PME) as the long-range electrostatic decomposition approach.^25^ All simulations were performed with GROMACS 2022.^15^

### Markov State Models

#### Feature Set Construction

We constructed and evaluated four feature representations: two *a priori* sets commonly used in MSM workflows and two custom sets tailored to the dominant motions observed in this system. Features were computed with PyEMMA (version 2.5.11).^26^

##### Preset Feature Sets

(i) Backbone torsions (BB): backbone dihedral angles were computed, yielding 308 features. (ii) Hydrophobic COM distances (Hydro COM): pairwise distances between side chain centers of mass (COMs) of hydrophobic residues were used as a reduced contact-based representation, yielding 903 features.

##### Custom Feature Sets

(iii) 94-feature set: starting from all pairwise distances between side chain COMs (3003 features), we applied TICA^27^ (20 ns lag time) to identify features correlated with the slow collective modes. We ranked features by their correlation with the first six TICA independent components (Supplementary Figures S1 and S2) and retained the most informative residue pairs while enforcing redundancy and locality filters (collapsing highly similar neighbors in a 3 × 3 index neighborhood and excluding terminal and near-neighbor pairs). This procedure yielded 92 distances, which were augmented by two angular descriptors capturing (a) the relative motion between helices α1 and α2 (C_α_ dihedral F86–F96–L129–A119) and (b) aromatic packing (angle between the ring planes of F92 and W134), giving a 94-dimensional feature set.

(iv) **18-feature set:** the 94-feat representation was used to build an exploratory MSM (TICA lag 20 ns, 30 dimensions; k-means clustering into 1000 microstates; MSM lag 100 ns).^26^ ,^28^ The implied time scales of this model showed substantial variance. Thus, we inspected representative structures from each metastable state and compared them with the set. Features were prioritized if they (i) showed clear, state-dependent shifts across the metastable ensemble, (ii) were consistent with the dominant TICA coordinates (i.e., strongly correlated with the slow modes), and (iii) reported on physically interpretable motions rather than local fluctuations. Applying these criteria yielded an 18-feature set comprising 7 COM distances (W134–F92, W134–L133, F92–L133, L77–F92, L77–L133, L77–W134, and L77–F86) and 9 C_α_–C_α_ distances (F86–K104, F86–N79, R95–H130, L129–A132, L129–W134, L133–R137, A132–P138, F86–L105, and F86–L134), plus the helix-motion dihedral and the F92–W134 aromatic angle (Figure 2B).

**Figure 2:**
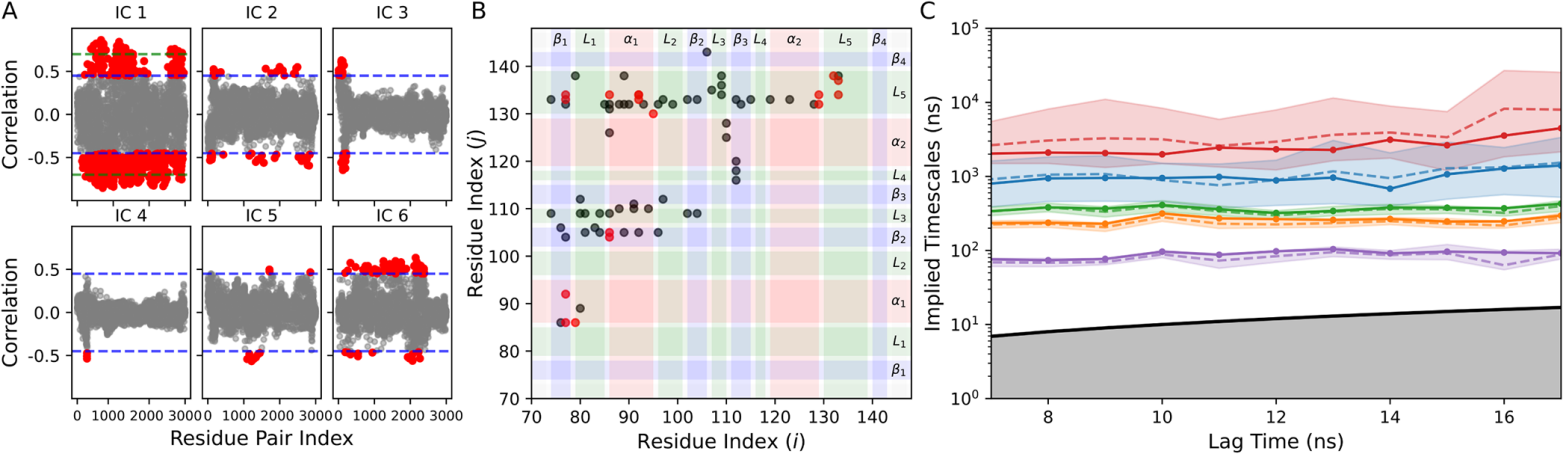
(A) Feature–TICA correlations of residue pairs for the first six independent components, shown as gray dots. Correlation values above 0.45 are shown in red, after the horizontal blue line threshold. The green line threshold indicates correlation values larger than 0.70. (B) Scatter plot of residue pairs. The full feature set of 92 residue pairs is shown in gray, while the 16 residue pairs from the final 18 feature set are shown in red. The protein secondary-structure elements are highlighted as β-strands (blue), α-helices (red), and loops (green). (C) Implied time scales of the five slowest dynamical processes. Dashed lines represent BHMSM sample means, while solid lines correspond to maximum-likelihood estimates. Shaded areas indicate 90% confidence intervals. The black line with the gray-shaded region denotes processes faster than the lag time.

#### Feature Set Evaluation

To evaluate the four feature sets, we assessed both their variational quality and the robustness of the resulting kinetics. First, we computed VAMP-2 scores^29^ over lag times from 2.5 to 100 ns (Supplementary Methods, Supplementary Figure S3). VAMP-2 provides a model-independent measure of how well a given representation approximates the slow dynamical modes; higher scores indicate that the features retain more kinetically relevant information. Across this lag-time range, the four feature sets exhibited comparable VAMP-2 scores, with the 94- and 18-feature sets showing a modest advantage at the longest lag times.

Because the TICA lag time sets the time separation used to estimate slow dynamical correlations (and larger lags reduce the number of statistically independent time pairs), we chose a short TICA lag time (2.5 ns) to maximize statistical robustness. This lag time was therefore used in subsequent analyses.

We further evaluated the discretization step by computing VAMP-2 scores for k-means clustering with 100, 250, 500, and 1000 microstates (Supplementary Figure S4). The scores were comparable across this range, indicating that model quality was not strongly sensitive to the number of clusters within these bounds. We therefore selected 100 microstates for subsequent analyses, as the simplest discretization that preserves VAMP-2 performance.

Based on the combination of (i) competitive VAMP-2 scores and (ii) reduced dimensionality, we selected the 18-feature representation for subsequent MSM construction and analysis.

#### Hidden Markov State Model

We constructed a Bayesian Hidden Markov state model (BHMSM) using the 18-feature representation. Model hyperparameters were evaluated by scanning the number of macrostates (4–8), the number of k-means microstates (100, 500, 1000 cluster centers), and the MSM lag time. Additional information can be found in the SI. We found that six macrostates, 100 microstates, and a model lag time of 10 ns provided a good compromise between model resolution and statistical robustness (Supplementary Figure S5, Supplementary Tables S1–S6).

The Chapman–Kolmogorov test was used to validate Markovianity by comparing model-predicted residence probabilities with those estimated from the data. As shown in Supplementary Figure S6, predicted and estimated probabilities agree closely, supporting the Markovianity of the six-state model. Alternative choices of the number of macrostates were also explored (Supplementary Figures S7–S10); however, these models either failed the CK test or yielded an overly coarse metastable decomposition.

Mean state lifetimes, relaxation time scales, and mean first passage times (MFPTs) were computed from the BHMSM. Uncertainties were estimated from the model’s posterior samples by fitting the resulting distributions with a Gaussian mixture model (GMM) using scikit-learn.^30^

For completeness, we computed implied time scales (ITSs) for MSMs built from the other three feature sets using the same number of macrostates (Supplementary Figure S11, Supplementary Tables S7–S8). While the ITSs were broadly similar across feature sets, only the 18-feature model showed consistent convergence (stable plateaus with lower variance across lag times) and passed the CK test, indicating a more robust separation of slow and fast processes.

#### Cavity Detection

Cavities were identified using MDpocket,^31^ which is based on the fpocket cavity detection algorithm.^32^ Fpocket detects potential binding cavity by generating an ensemble of α-spheres that capture the local geometry of the protein. Each α-sphere represents a void region tangent to four protein atoms, providing a geometric approximation of the cavity shape and size. To identify the transient cavity, we used an isovalue of 2, as recommended by MDpocket. The isovalue corresponds to the number of α-sphere centers within an 8 Å^3^ grid cube per snapshot of the MD trajectory. Thus, a selected grid point must contain at least two α-sphere centers within this volume to be considered part of a cavity. The cavities were characterized by their volume, calculated from the total number and spatial distribution of α-spheres, and by the mean local hydrophobic density, which quantifies the clustering of hydrophobic α-spheres within each cavity. The mean local hydrophobic density evaluates whether a cavity contains locally concentrated hydrophobic regions. For each apolar α-sphere, neighboring apolar α-spheres are identified by detecting overlaps between them. The total number of such apolar–apolar overlaps is summed over the entire cavity and divided by the total number of apolar α-spheres, yielding the mean local hydrophobic density.^33^

## Results

### Feature Selection and MSM Construction

To capture motions relevant to allosteric communication within the *h*TRMT2A RRM, we initially explored standard feature representations commonly used in MSM workflows, namely backbone torsions (BB) and contact-based COM distance features restricted to hydrophobic residues (Hydro COM). While these preset representations yielded competitive variational scores (Supplementary Figure S3), kinetic validation showed that the resulting models were comparatively sensitive to discretization and lag-time choice, motivating a more targeted representation focused on the dominant slow rearrangements.

We therefore constructed a custom distance-based feature set by enumerating all inter-residue side chain COM distances across the domain and using TICA to identify features aligned with the slow dynamical modes (Figure 2A). Inspection of the leading independent components showed that IC 1, 2, 5, and 6 describe concerted rearrangements spanning the helical and β-sheet faces, whereas IC 3 and 4 are dominated by terminal fluctuations (Supplementary Figure S2). We therefore prioritized features correlated with IC 1, 2, 5, and 6 and pruned the distance list using redundancy and locality filters (Supplementary Figure S1), yielding 92 informative residue-pair distances (Figure 2B, gray dots). These distances are enriched in contacts involving loop 5, couplings between β2/β3 and α1 (including adjacent loops), and packing interactions linking the helical surface to the RNP sheet, consistent with helical-surface/RNP-sheet coupling. We augmented the 92 distances with a helix-reorientation dihedral and the F92–W134 aromatic packing angle, yielding a 94-feature representation.

An initial MSM built from this representation resolved six provisional macrostates. However, the implied time scales (ITSs) did not converge across lag times, indicating insufficient stability, likely due to the high dimensionality of the system and suboptimal discretization. We therefore examined representative structures from each macrostate and identified key slow motions localized around loop 5 and the α1-helix (Figure 2B). In particular, the dominant conformational changes consistently involved residues W134, L133, and F92, together with coordinated motions of the α-helical region.

To assess whether these structural signatures were specific to the reduced representation, we also performed preliminary BHMSM analyses using the less targeted feature sets, namely BB and Hydro COM. Although these higher-dimensional models showed poorer ITSs convergence and lower kinetic stability than the optimized reduced model (Supplementary Figure S12), they were still qualitatively informative. In particular, the resulting macrostates repeatedly highlighted structural differences involving W134, L133, and F92, together with loop 5 dynamics and α-helix rearrangements (Supplementary Figures S13–S14), in line with the previous observations. The role of L133 was less clearly resolved in the BB representation, consistent with the fact that its contribution is more directly captured when side chain information is included explicitly. Taken together, these observations indicate that these structural motifs recur across other broader feature representations, justifying their choice.

This analysis guided a targeted refinement to 18 kinetically informative features that maximized kinetic resolution while preserving residue-level interpretability. The final feature set comprises COM distances (W134–F92, W134–L133, F92–L133, L77–F92, L77–L133, L77–W134, and L77–F86), Cα–Cα distances (F86–K104, F86–N79, R95–H130, L129–A132, L129–W134, L133–R137, A132–P138, F86–L105, and F86–L134), a dihedral angle describing the relative motion between helices, and the angle between the planes of F92 and W134 (Figure 2B, red dots). This reduced representation emerged from iterative pruning constrained by the previous observations as well as variational scoring.

Using this 18-feature set, we trained a BHMSM. The implied time scales (Figure 2C), together with Chapman–Kolmogorov validation (Supplementary Figure S6), supported the Markov assumption at the chosen lag time and provided the basis for extracting kinetic information from this model.

### Structural Properties of the Six Metastable States in *h*TRMT2A

The optimized BHMSM resolved six metastable states (S1–S6) describing the slow dynamics of the *h*TRMT2A RRM. Among these, two states dominate the equilibrium ensemble: S6 (62% occupancy) and S5 (24%), together accounting for approximately 86% of the conformational ensemble (Figure 3A, Supplementary Table S9). Both states closely resemble the crystal structure, exhibiting the lowest heavy-atom RMSD values. The remaining four states (S1–S4) are sparsely populated, each contributing 2–5% of the total equilibrium probability. To elucidate the structural basis of these differences, we compared the secondary-structure composition, residue interactions involving loop 5 and the α2-helix, the relative orientation of the α1/α2-helices, and local flexibility across all six states (Figure 3B–D).

**Figure 3:**
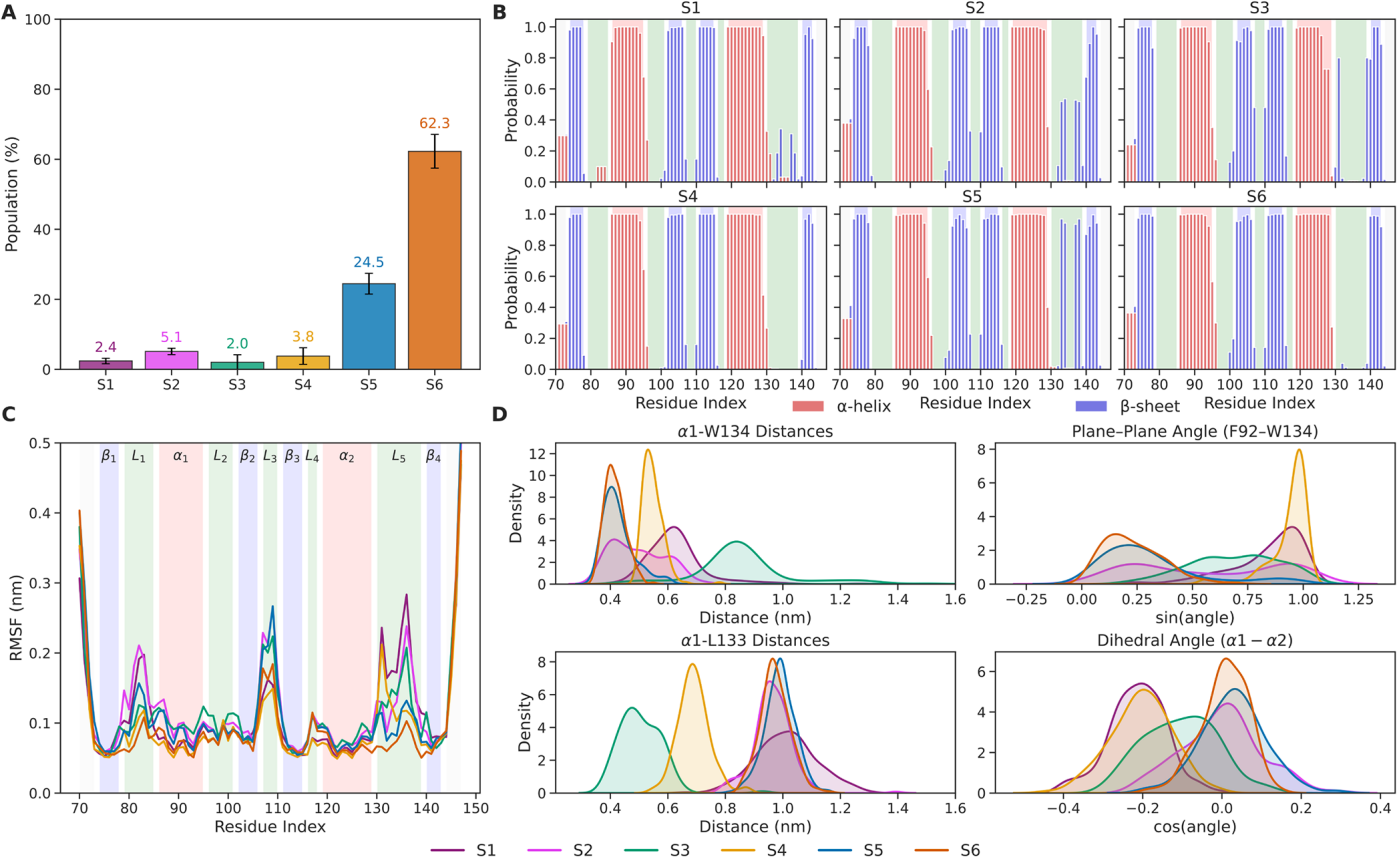
(A) Stationary populations of the metastable states. Error bars represent the standard deviation. (B) Secondary structure probabilities per residue for the metastable states. Bars indicate the cumulative probabilities of α-helix (blue) and β-sheet (red) content, the remaining fraction up to 1.0 corresponds to random coil. (C) Cα root-mean-square fluctuations (RMSFs) of the metastable states, calculated after alignment to the crystal structure. (D) Distributions of structural features for the metastable states: The side chain COM distances between F92–W134 (top, left), the angle between the planes of W92 and W134 (top, right), the side chain COM distances between F92–W133 (bottom, left) and the dihedral angle describing helix motion (bottom, right). The color code is used for the six metastable states in (A), (C), and (D): S1 (sky blue), S2 (pink), S3 (green), S4 (orange), S5 (blue), and S6 (red). Protein secondary-structure elements in (B) and (C) are highlighted as β-strands (blue), α-helices (red), and loops (green).

The metastable states show notable differences in their secondary-structure content (Figure 3B). Taking the most populated state, S6, as a reference, S5 exhibits additional β-structure within loop 5 (residues 133–134 and 137–138), consistent with a slightly more rigid conformation. This observation suggests that S5 and S6 interconvert through localized β-sheet formation in loop 5. The third most abundant state, S2, shows a similar but less pronounced rigidization in this region, indicating partial stabilization of the same structural motif.

The least populated states (S1, S3, S4) display broader rearrangements involving both the α2-helix and β-sheet elements. In S4, β-sheet structure is partially lost at residues 78 (β1), 100 (β2), and 116 (β3), accompanied by a slight extension of the α2-helix near residue H130. S1 shows a comparable pattern of β-sheet loss but with the additional ordering within loop 5 as described before. The α2-helix content in S1 also extends through residues 130–131. Conversely, S3 gains β-sheet content at residues 107 (β2), 110 (β3), 131 (loop 5), and 139 (β4), while its α2-helical segment shortens, losing helical propensity at residue 129. Overall, these patterns indicate that loop 5 and the C-terminal portion of the α2-helix are key structural hotspots modulating the conformational heterogeneity of the *h*TRMT2A RRM.

The root-mean-square fluctuations (RMSF) (Figure 3C) show that deviations from the crystal structure are most pronounced in loop 1, loop 3, and loop 5. States S6 and S5 are the most rigid, whereas the remaining states display elevated flexibility in these regions. Notably, residues 132–134 within loop 5 do not exhibit the same increase in mobility as neighboring residues, suggesting the presence of local stabilizing interactions that anchor this segment.

The gain of secondary structure within loop 5, together with its localized rigidity at residues 132–134, prompted us to investigate the interactions responsible for this stabilization. In the most populated metastable states, S6 and S5, W134 remains buried in close contact with F92 from the α1-helix, forming a nearly parallel π–π stacking arrangement (Figure 2D, upper row). Given this characteristic, we thus refer to the F92/W134 as an aromatic lock. This lock, quantified through both inter-residue distances and ring-plane orientation angles, keeps a surrounding compact hydrophobic core that maintains a rigid conformation on the *h*TRMT2A RRM. In S2, the aromatic lock becomes slightly disrupted due to the formation of the transient β-sheet content within loop 5, as reflected in the increased inter-residue distances and a wider distribution of plane orientations. This intermediate state thus alternates between mixed π–π and hydrophobic interactions.

Less populated states exhibit distinct rearrangements of this aromatic lock. In S4, W134 tilts away from F92, adopting a T-shaped ring orientation. Concomitantly, L133 moves inward toward F92, likely compensating for the change of aromatic stacking by shielding the hydrophobic core from solvent exposure. In S3, the shift becomes complete: W134 disengages entirely, and L133 replaces it, effectively redefining this interaction as purely hydrophobic (Figure 2D, lower left). Conversely, in S1, W134 drifts away from F92 without replacement by nearby residues, leaving the site exposed and highly flexible, consistent with its low population (Figure 2D, lower right). In all these states, we observed the formation of a small cavity in place of the broken aromatic lock, depending on the degree of displacement of W134, as suggested by Margreiter et al.^9^

The dihedral angle between the α1- and α2-helices mirrors the hydrophobic rearrangements described above. In the closed states S6 and S5, the helices adopt a nearly perpendicular orientation that maintains a compact fold, as quantified by their lower radius of gyration (Supplementary Table S9), with S2 retains a comparable geometry with minor deviations. In contrast, S3, S4, and S1 exhibit a shift of the α2-helix accompanied by the concerted burial of residue F86, which is solvent-exposed in the closed conformations. This rearrangement not only extends the hydrophobic network toward the domain core but also increases the distance between the RNP region and the helices, creating a secondary cavity, only present in these high-energy states.

Together, these observations indicate that the aromatic and hydrophobic interactions within loop 5—particularly those involving W134, F92, and L133—act as a molecular switch that stabilizes the interhelical orientation. The dynamics of this aromatic lock control both the formation of cavities within the structure as well as the solvent exposure of F86. F86 thus emerges as a reporter residue nearby the RNP1/2 β-sheet region, reflecting the state of the aromatic lock and linking conformational changes in loop 5 to alterations at the RNA-binding surface.

### Macrostates in *h*TRMT2A Seem to Be Stabilized through Defect Formation in the α2-Helix

The analysis above established that the motion of residues within loop 5 plays a key role in shaping the conformational landscape. However, this loop does not operate in isolation: its conformational changes are tightly coupled to the structural plasticity of the adjacent α2-helix, which can partially unfold to provide the mechanical leverage required for rearrangement of the hydrophobic cluster.

In this way, the α2-helix adopts three discrete conformational states (Figure 4A). The most populated form, designated α2-H1 and observed in macrostates S6, S5, and S2, extends the helix to residue 130. Transitions to higher-energy conformations involve either loss of residue 130 from the helical register (α2-H2; S3 and S4) or incorporation of residue 131 into the helix (α2-H3; S1). These assignments are corroborated by changes in local backbone angles and diagnostic inter-residue distances (Figure 4B).

**Figure 4:**
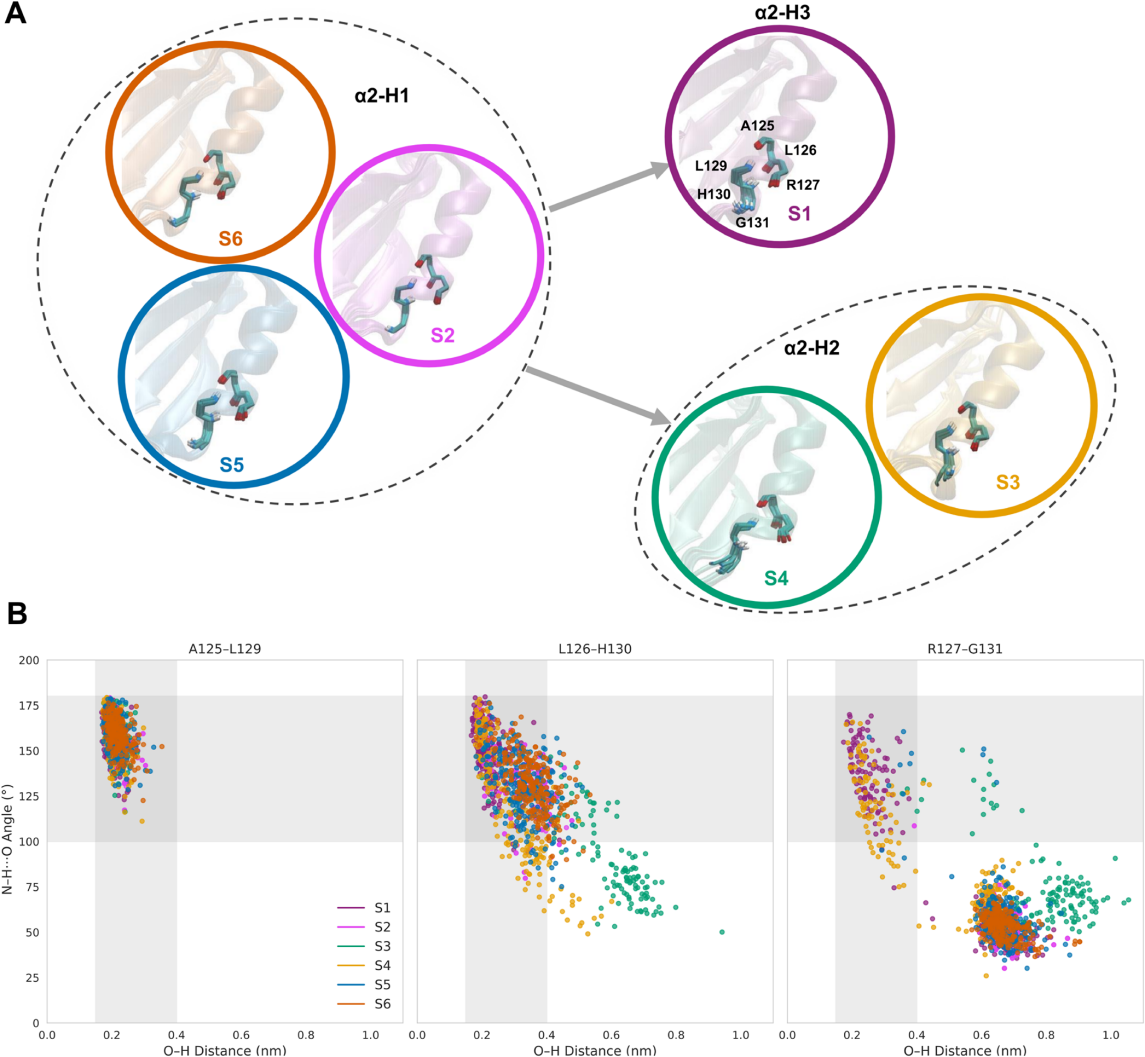
(A) Three conformational states of the α2-helix (α2-H1, α2-H2, and α2-H3). The protein is shown in cartoon representation. Hydrogen-bonding atoms of residues A125–L129, L126–H130, and R127–G131 within the α2-helix are displayed as sticks, and residue labels are shown in the α2-H2 state. Hydrogen, oxygen, and nitrogen atoms are shown in white, red, and blue, respectively. (B) Scatter plots of H, O distances and N–H, O angles describing hydrogen-bond formation between the NH group and backbone oxygen atoms of residues A125–L129, L126–H130, and R127–G131 across the metastable states. The gray-shaded region indicates the geometric criteria used to define hydrogen bonds, corresponding to H, O distances of 0.15–0.40 nm and N–H, O angles between 100° and 180°.

Within a soft-matter perspective, the partial unwinding of α2-which modulates loop 5 motion—constitutes a *connectivity defect*: a local break in the continuity of an otherwise ordered elastic element.^34–37^ A useful reference state is a perfectly formed α2-helix with a continuous *i* → *i* + 4 hydrogen-bond network, expected to occupy a deep, narrow energy well separated from alternative conformations by high activation barriers. Indeed, AlphaFold2 predictions indicate that residues 119–134 have strong intrinsic helical propensity (Supplementary Figure S15). Yet if fully helical in the protein context, the system would become overly rigid and unable to execute the conformational transitions required for allosteric communication.

This finely tuned structural plasticity of α2, together with loop 5 motion, defines a coupled hinge–gate mechanism. Partial unfolding of α2 modulates loop 5 positioning and thereby tunes accessibility of the cavity adjacent to the RNP face. Thus, the connectivity defect in α2 is not a passive mechanical flaw but an active, tunable control element that couples local instability to global functional adaptability. The interplay between these elements underlies the protein allosteric regulation and contributes to its overall thermodynamic stability.

### Cavity Opening in the *h*TRMT2A RRM is Driven by Reorganization of Aromatic Residues W134 or F86

The structural rearrangement surrounding the RNP region in the high-energy conformations facilitates the formation of cavities in the *h*TRMT2A RRM. Two cavities were initially observed: one in the opening of the aromatic lock due to exposure of W134, and another one due to burial of residue F86, near the RNP. To evaluate other possible cavities and characterize them, we employed MDpocket.^31,32^ Through this approach, we were able to identify and characterize three major cavities: (i) near the aromatic lock F92/W134, (ii) adjacent to F86, and (iii) along the RNP face (Figure 5A). We evaluated these cavities based on their volumes and mean local hydrophobic density; parameters that have been shown to correlate strongly with pocket druggability.^33^ To further support our observations, druggability scores and cavity volumes were computed using DoGSiteScorer (Supplementary Table S10) for the top clusters of each state.^38,39^ The identified pocket locations for the top clusters closely match the cavities described here.

**Figure 5:**
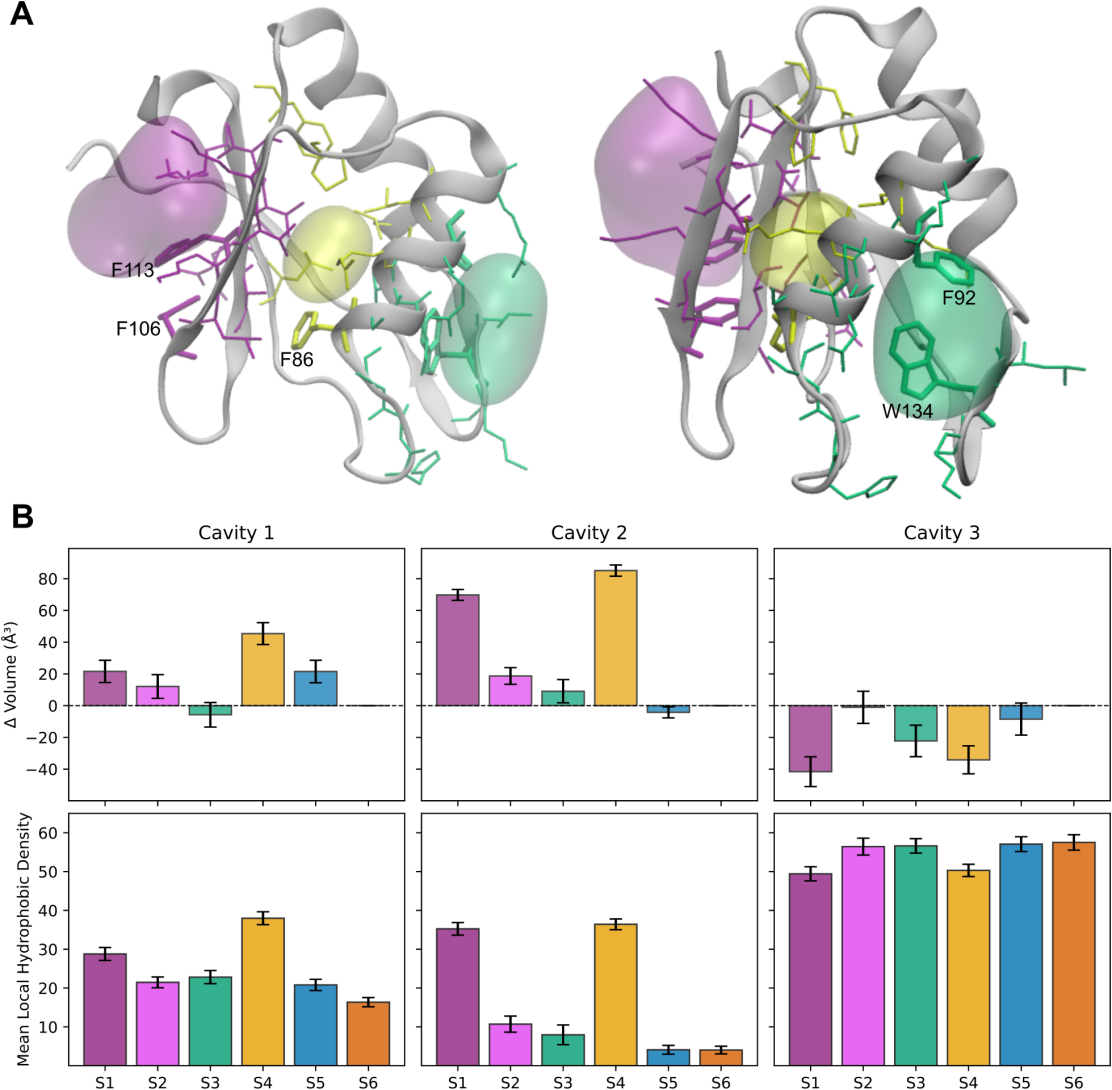
(A) Cavities identified using MDpocket. Cavities are shown as surfaces in green (Cavity 1), yellow (Cavity 2), and purple (Cavity 3). Residues defining each cavity are displayed as sticks in the corresponding cavity color, and the protein representative structure (cluster center of open state S1) is shown in cartoon representation. (B) Relative changes in cavity volume (with respect to S6) and mean local hydrophobic density across the metastable states. Error bars represent the standard error.

The first cavity, located near the aromatic lock and primarily involving residues from the α1-helix, loops 1 and 5, includes H83–S85 (loop 1), S87–V89, R91, F92, R95 (α1-helix), and A132–K135 (loop 5). This cavity exhibits slightly larger volumes in the open conformations (S1 and S4) compared to the closed ones (Figure 5B and Supplementary Table S11). Notably, the mean local hydrophobic density is also higher in the open conformations, reflecting the outward movement of W134 away from F92, which exposes part of the hydrophobic core to the solvent and contributes to cavity expansion. Previous work suggests that several molecules with phenyl groups could interact with the predicted cavity, as their aromatic rings could potentially π-stack with F92.^9^

The second cavity, located adjacent to F86, primarily involves residues from the α1-helix (F86, V89, R90, L93), β2-strand (T103, L104), and β3-strand (A112, V114, F116). This cavity is detected exclusively in the open conformations (S1 and S4). Its formation is driven by a shift of the α2-helix, burial of residue F86, and an increased separation between the RNP region and surrounding helices. This structural change is evident from the increased distance between F86 in the α1-helix and F106 in the β2-strand, from 1.022 ± 0.081 nm in the closed conformations (S6 and S5) to 1.205 ± 0.057 nm in the open conformations (S1 and S4), which collectively facilitate cavity formation. Both the cavity volume and mean local hydrophobic density (Figure 5B) are significantly higher in the open states, indicating enhanced cavity accessibility and hydrophobic character.

The third cavity is located on the RNA binding surface, defined primarily by the β-sheet region. This area is enriched in aromatic residues, particularly F106 and F113, which form π-stacking interactions with RNA^7,8^ and also includes C111, which has been experimentally shown to participate in RNA binding.^5^ Interestingly, a slight decrease in local hydrophobic density is observed in the open conformations (S1 and S4), coinciding with the inward movement of F86.

### Microsecond-Scale Transitions Underlie Cavity Formation in the *h*TRMT2A RRM

Having established a hinge–gate mechanism in which loop 5 motion is coupled to partial unfolding of the α2-helix, which then leads to the formation of the cavities in RRM *h*TRMT2A, we asked *how* the hydrophobic residues (W134, L133, F92, and F86) lead the transition sequence between said macrostates. For this, we applied transition path theory (TPT)^40,41^ to the BHMSM. TPT yielded the dominant transition pathways among the metastable states (Figure 6A, Table 1, and Supplementary Table S12), as well as state lifetimes (Figure 6B, Supplementary Table S9) and slowest relaxation time scales (Figure 6C, Supplementary Table S13).

**Figure 6:**
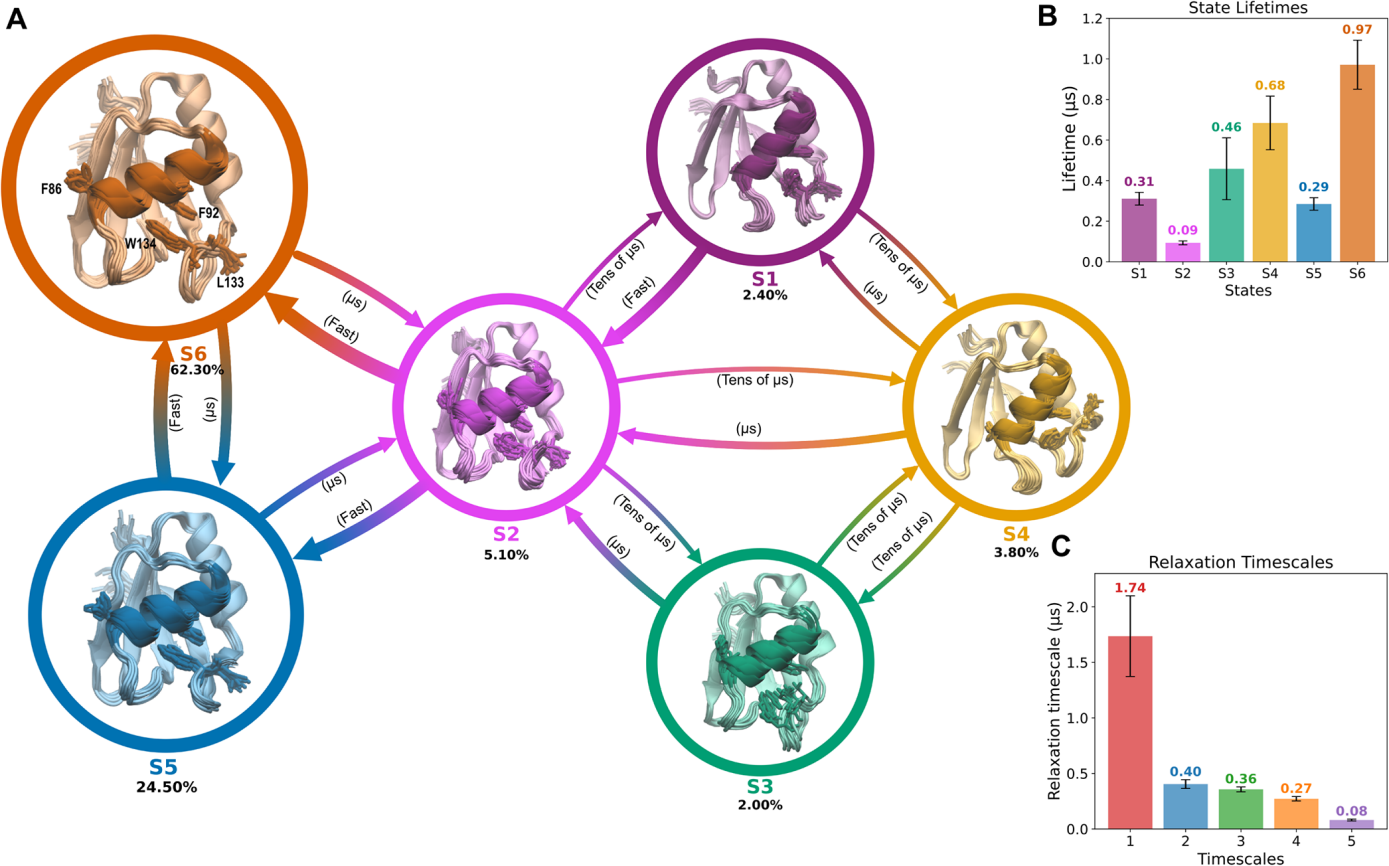
(A) Network diagram of the metastable states and their stationary populations. States are represented as circles, and their populations are shown. Arrows indicate transition rates, annotated with mean first-passage times and their standard deviations. MFPTs are categorized as Fast (<1 μs), μs (μs range), and Tens of μs (>10 μs). Representative conformations of each metastable state are shown as cartoons, with residues F86, F92, L133, and W134 highlighted as sticks. α1-helix is also highlighted in the cartoon depiction. For clarity, zoom-in views for all metastable states are provided in the Supporting Information (Supplementary Figure S16). (B) Lifetimes of the metastable states. (C) Slowest relaxation time scales of the model. For both plots, error bars denote standard deviations.

**Table 1:** Transition Pathways (up to ∼90% of the Total Flux)

Paths	Percentage (%)
S6 → S5 → S2 → S1	67.12
S6 → S2 → S1	20.21
S6 → S2 → S3 → S4 → S1	5.12

Mean first-passage time (MFPT) analysis revealed that transitions between metastable states span hundreds of nanoseconds to tens of microseconds. State S2 emerges as the principal intermediate bridging the closed F92/W134 core (S6/S5) and the open one (S1/S3/S4). Accordingly, the transition network can be organized into four classes: (i) interconversion within the closed ensemble (S6 ↔ S5), (ii) exchange between S6/S5 and the hub state S2, (iii) transitions between S2 and the open states (S1, S3, S4), and (iv) interconversion among the open states.

Delving further into the transition network: (i) First, transitions between the lowest-energy states S6 and S5 are relatively fast, consistent with rapid formation/disruption of the β-structure within loop 5. (ii) Moving forward, the exchange between S6/S5 and S2 involves weakening of the F92–W134 aromatic lock. These processes occur on the order of microseconds and are readily reversible. Across S6, S5, and S2, no large-scale rearrangements are evident, as reflected by minimal changes in the α1-helix tilt and the solvent exposure of F86 (Supplementary Figure S17A). (iii) In contrast, transitions from S2 to the open-lock states are markedly slower, reflecting large-scale structural reorganization. The fastest is S2 → S1 (∼17 μs), characterized by opening of the F92–W134 lock, tilting of the α1-helix, and burial of F86. Paths to S3 or S4 from S2 are slower (∼36–42 μs) and involve partial or complete α1 tilting with corresponding partial or complete burial of F86. The slowest step is S2 → S3, which rewires the lock by substituting W134 with L133 and yields only partial burial of F86. (iv) Finally, interconversion among the high-energy states proceeds via S4, with MFPTs values ranging from ∼6 to 28 μs.

Consistent with this network view, S2 is the shortest-lived state (Figure 6B), rapidly giving rise either to the closed-lock states (S6/S5) or to the open ones (S1/S3/S4). Within the closed-lock ensemble, S5 is also short-lived and quickly returns to S6 as loop 5 β-structure melts. Among the open states, S4 is the longest-lived. These MFPTs estimates are therefore most robustly interpreted in terms of the relative ordering of state lifetimes and pathway structure, rather than as quantitatively calibrated absolute kinetics.

Transition-path theory (TPT) further shows that flux from the most stable closed-lock state (S6) to the most energetic open state (S1) is concentrated in two dominant pathways that account for 87% of the total flux. The major pathway (67%) follows S6 → S5 → S2 → S1: loop 5 first forms β-structure (S5), which then destabilizes the aromatic lock, allowing α1 tilting and promoting burial of F86 (S2), culminating in S1. A secondary pathway (20%) bypasses S5, proceeding through S6 → S2 → S1, indicating that loop 5 β-structure formation is not strictly required for opening, even if it stabilizes the preferred multistep route.

The MFPTs values reported here remain model- and simulation-dependent and may vary with different choices of hyperparameters. In addition, hydrogen mass repartitioning can systematically alter absolute kinetic rates, although previous studies have shown that relative pathway ordering and the underlying thermodynamics of conformational transitions are generally preserved.^42^ Accordingly, we interpret the MFPTs values comparatively, emphasizing relative time scales and pathway structure rather than absolute kinetic rates.

In summary, the kinetic analysis highlights a coordinated mechanism with a clear time scale separation: sub- to few-microsecond local rearrangements within the closed ensemble versus tens-of-microseconds transitions that remodel the hydrophobic network and give rise to diverse cavity formation.

## Discussion

We investigated the conformational dynamics of the *h*TRMT2A RRM using atomistic MD simulations. MD simulations typically requires a longer time to converge, undersampling interesting conformations such as higher-energy metastable states and transient (cryptic) pockets. To address this sampling gap, we employed Markov state models (MSMs), which stitch together many trajectories to reconstruct long-time scale kinetics and expose rare events within a single framework.^12^ While alternative approaches, such as network- or graph-based methods, are powerful tools for identifying residue–residue communication pathways and mapping allosteric networks^43,44^ MSMs provide a complementary perspective by delivering an explicit kinetic and thermodynamic description of the conformational ensemble, rather than a purely static or time-averaged representation.

As outlined in the Introduction, hydrophobic and aromatic residues are indispensable to RRM function, especially for nucleic-acid recognition.^7,8^ Our results point toward a central role for loop 5 in regulating conformational switching among *h*TRMT2A RRM states. In low-energy metastable states S6 and S5, the aromatic side chain of W134 orientates inward toward the α1 helix and stacks with F92, minimizing solvent exposure. This π–π interaction forms an *aromatic lock* that stabilizes a compact ensemble, consistent with the reduced solvent-accessible surface area (SASA) of these states (Supplementary Figure S17B). In higher-energy states (S1, S3, S4), disruption of the F92–W134 interaction breaks the lock, allowing loop 5 to move and enlarging cavity 1 (Figure 5A). Concomitantly, other residues become more solvent-exposed (Supplementary Figure S17A). This transition is coupled to an outward displacement of the α1 helix, which moves away from the core to accommodate an expansion of cavity 2 and the burial of F86 (Figure 5A).

Loop flexibility depends strongly on sequence composition. Loop 5 comprises A132–L133–W134–R135–G136–R137–P138–L139. While leucine and arginine are common in dynamic loops, the presence of a bulky, hydrophobic tryptophan at position 134 is notable, as tryptophan is underrepresented in loop regions.^45^ Nevertheless, W134 (or a phenylalanine at the equivalent position) is conserved across TRMT2A homologues, underscoring its functional importance—a conclusion that aligns with our locking mechanism.

State-dependent cavity formation points to plausible interaction sites for molecular partners. Prior work suggested that motion of W134 can reveal a cryptic pocket.^9^ Our MSM indicates that the opening of cavity 1 occurs on the order of tens of microseconds. A second site (cavity 2) emerges from the inward movement of α1 and the burial of F86. Among the observed conformational states, S1 and S4—where the F92–W134 lock is broken without compensatory packing and F86 is buried—present the most promising geometries for ligand interaction at said cavities.

The exposure of aromatic side chains is a key determinant of RNA recognition by RRMs.^7^ In the crystallized structures of the *h*TRMT2A RRM, two phenylalanines reside on RNP1, but only F113 consistently presents its side chain to solvent; F116 remains buried in the hydrophobic core. On RNP2, F73 is likewise buried, which likely limits its direct role in base stacking. These observations suggest that additional residues may be needed to stabilize nucleobases during binding. In this context, our model underscores a potential role for F86 in RNA recognition. In the most stable states (S6, S5, S2), F86 is solvent-exposed. Transitions to conformations in which F86 becomes buried require partial unwinding of the α2 helix and a register shift of α1—an energetically costly move. We therefore hypothesize that solvent exposure of F86 facilitates initial RNA capture, whereas its burial in higher-energy states constitutes a self-inhibitory switch. Consistent with this model, the volume and hydrophobicity of cavity 3 decrease when F86 is internalized, reducing the likelihood of accommodating nucleobases.

As an outlook, our model suggest potentially testable predictions for experimental validation: (i) *Lock disruption:* Mutations such as W134A/F and F92A can potentially destabilize S6/S5, shifting populations toward open states; L133A/V may also suppress the S3 ″alternative lock.″ (ii) *Helix propensity tuning:* Helix-favoring substitutions at 129–131 could bias toward closed states and reduce pocket opening, whereas helix-breaking substitutions (e.g., Pro/Gly at 129–131) should favor opening. These effects can be prospectively probed via RNA-binding assays (ITC/SPR) and as well as spectroscopy (NMR, smFRET), testing whether reduced binding correlates with stabilized open states (and vice versa).

## Conclusions

By combining atomistic MD simulations with MSMs, we resolve the conformational landscape and allosteric coupling of the *h*TRMT2A RRM. Our results support a coherent mechanism in which a hydrophobic cluster centered on L133/W134–F92, mechanically coupled to a soft, defect-like segment in α2, modulates the position of α1, the exposure of F86, and the breathing of loop 5. This hinge–gate architecture toggles between compact (locked) and open ensembles and remodels solvent exposure across the RNA-binding surface.

Our analysis focuses on the RNA-free RRM. Model choices (force field, water model, protonation), feature selection/discretization, and the absence of post-translational modifications and the C-terminal catalytic domain may influence kinetics and state populations. Extending the MSM to RNA-bound simulations and to full-length *h*TRMT2A will be important to test how RNA engagement feeds back onto the hinge–gate.

The state-resolved pockets and switching residues provide concrete entry points for ligand discovery. In particular, *open-state stabilizers* that wedge near loop 5/α1, should disrupt the F92–W134 lock, or stabilize the buried state of F86, thus modulating TRMT2A activity in polyQ disease biology.

In sum, *h*TRMT2A RRM leverages a connectivity-defect–enabled hinge in α2 and a hydrophobic microswitch in loop 5 to coordinate distant surfaces and regulate cryptic-pocket opening. This mechanism explains how local instability produces global adaptability and offers actionable targets for allosteric control in polyQ contexts.

## Data and Software Availability

Molecular structures of the six metastable states in PDB format, as well as Jupyter Notebooks with the code used for this work, can be found in a GitHub repository: https://github.com/palominohernandez/TRMT2A-2025.

## Supplementary Material



## Notes

Supplementary Methods: a detailed description of VAMP-2 scoring analysis, dimensionality reduction using TICA, selection of optimal lag time, microstate discretization using k-means clustering, and construction and validation of BHMSMs; Table S1: state populations for 4-macrostate BHMSM models across different clustering resolutions and lag times; Table S2: relaxation time scales for 4-macrostate BHMSM models; Table S3: state populations for 5-macrostate BHMSM models across clustering resolutions and lag times; Table S4: relaxation time scales for 5-macrostate BHMSM models; Table S5: state populations for 6-macrostate BHMSM models across clustering resolutions and lag times; Table S6: relaxation time scales for 6-macrostate BHMSM models; Table S7: comparison of state populations across different feature sets (18 features, 94 features, backbone torsions, hydrophobic COM distances); Table S8: comparison of relaxation time scales across different feature sets; Table S9: summary of thermodynamic and structural properties of metastable states, including stationary probabilities, free energies, lifetimes, RMSD, and radius of gyration; Table S10: druggability scores and cavity volumes computed using DoGSiteScorer for top-ranked clusters across metastable states; Table S11: cavity properties across metastable states, including volumes, hydrophobic densities, and occupancies; Table S12: MFPTs between metastable states with uncertainties; Table S13: slowest relaxation time scales of the six-state model; Figure S1: feature–TICA correlations of residue pairs for the first 40 independent components; Figure S2: matrix representation of feature–TICA correlations across the first 40 independent components; Figure S3: comparison of VAMP-2 scores across feature sets using cross-validation; Figure S4: VAMP-2 scores as a function of the number of k-means clusters for different feature representations; Figure S5: implied time scales of the slowest dynamical processes for the 18-feature BHMSM model across different microstates and macrostates; Figures S6–S10: Chapman–Kolmogorov validation of the six, four, five, seven, and eight-state BHMSM models; Figure S11: comparison of implied time scales across different feature sets; Figure S12: implied time scale analysis for hydrophobic COM and backbone torsion feature sets across multiple state decompositions; Figure S13: distributions of key structural features across metastable states for hydrophobic COM features; Figure S14: distributions of key structural features across metastable states for backbone torsion features; Figure S15: AlphaFold2 structural predictions for residues 119–134; Figure S16: representative conformations of metastable states highlighting key residues and secondary structure elements; Figure S17: analysis of SASA differences across metastable states; Figure S18: spectral gap analysis of MSM implied time scales (PDF)
