# Supplementary material for: A Hydrophobic Cluster Modulates Long-Range Allostery in the TRMT2A RNA Recognition Motif
Source: J Chem Inf Model. 2026 May 12;66(10):5978–89. doi: 10.1021/acs.jcim.5c02753 (PMC13213904; doi:10.1021/acs.jcim.5c02753)
Supplement: Supplementary file 1 [file ci5c02753_si_001.pdf]

Supporting information: A hydrophobic cluster modulates long-range allostery in the TRMT2A RNA recognition motif

Mohammed Khaled[1,†], Lisa Johannknecht[1], and Oscar Palomino-Hernandez*[1,2,†]

[1]Department of Chemistry, Johannes Gutenberg University Mainz, Duesbergweg 10-14, D-55128 Mainz, Germany
[2]Institute for Quantitative and Computational Biosciences (IQCB), Johannes Gutenberg University Mainz, Johannes-von-Müller-Weg 6, D-55128 Mainz, Germany
[†]These authors contributed equally.

*Email: opalomin@uni-mainz.de

Supplementary Methods

Markov State Models

VAMP2

To compare the four feature representations: hydrophobic residue center-of-mass (COM) distances (903 features), the 94-feature set, the reduced 18-feature set, and backbone torsions (308 features)—their variational quality was systematically evaluated using VAMP-2 scores for each feature set. A five-fold cross-validation scheme was employed, in which 100 trajectories (66%) were used for training and 50 trajectories (33%) for testing in each split (Figure S3). The VAMP-2 Scores were calculated at lag times of 2.5, 25, 50, and 100ns and in two dimensions.

Dimensionality Reduction and Discretization

Time-lagged independent component analysis (TICA) was applied to the 150 trajectories (each 1 μs) to reduce the dimensionality of the feature space. A lag time of 2.5 ns (50 simulation steps) was used, and the trajectories were projected onto the two dominant time-lagged independent components. To determine an appropriate discretization, VAMP-2 scores were computed as a function of the number of clusters using five independent k-means++ initializations with 200 iterations. Convergence was observed at 100 microstates (Figure S4), which were subsequently used for k-means clustering and MSM construction.

Hidden Markov state model

The implied timescale spectra derived from the Markov state model (MSM) were analyzed to evaluate the choice of microstate discretization (Figure S18). Using a TICA lag time of 2.5 ns and an MSM lag time of up to 100 μs, the timescale separation revealed four pronounced gaps between the dominant slow processes, supporting the selected level of state resolution. Based on this analysis, a six-macrostate Bayesian hidden Markov state model (BHMSM) with a lag time of 10 ns (200 simulation steps) was chosen. This model exhibited clear separation of the slowest dynamical processes while maintaining a detailed yet statistically robust representation of the conformational landscape.

The BHMSM framework employs Gibbs sampling to infer posterior distributions over hidden states and transition probabilities. The model was initialized with six metastable states, and mixed priors were applied to both the initial state distribution and the transition matrix. Posterior sampling yielded 5,000 transition matrix realizations, enabling estimation of statistical uncertainties in kinetic observables. For structural

characterization, 300 representative conformations were extracted from each macrostate to describe state-specific structural features.

Supplementary Tables

Table S1: State populations for 4-macrostate BHMSM models.

K-means	HMM Lag (ns)	S1	S2	S3	S4
100	8	4.64±5.55	7.53±3.68	9.68±1.68	78.15±6.51
	10	4.94±5.37	8.31±4.51	7.55±1.46	79.21±7.06
500	8	4.83±6.02	6.62±3.19	9.44±1.56	79.11±6.53
	10	4.63±5.12	9.82±5.36	9.46±1.60	76.09±7.34
1000	8	5.19±6.38	7.68±4.13	9.07±1.60	78.06±7.22
	10	4.39±6.46	10.88±1.90	4.68±1.79	80.05±6.23

Table S2: Relaxation timescales (µs) for 4-macrostate BHMSM models.

K-means	HMM Lag (ns)	t1	t2	t3
100	8	2.49±2.19	0.85±0.36	0.20±0.02
	10	2.39±1.77	0.73±0.44	0.23±0.02
500	8	2.52±2.59	0.84±0.32	0.21±0.02
	10	2.71±1.80	0.85±0.46	0.20±0.02
1000	8	2.56±2.26	0.90±0.40	0.20±0.02
	10	2.45±4.15	0.45±0.19	0.15±0.03

Table S3: State populations for 5-macrostate BHMSM models.

K-means	HMM Lag (ns)	S1	S2	S3	S4	S5
100	8	5.86±6.90	6.51±3.57	5.97±1.23	27.04±3.37	54.62±5.84
	10	4.11±4.66	6.65±3.98	5.45±1.19	28.24±3.16	55.56±5.29
500	8	5.62±6.90	5.70±2.93	6.61±1.31	29.92±3.34	52.15±5.35
	10	4.61±5.22	7.76±4.10	7.18±1.37	29.48±3.20	50.97±5.13
1000	8	5.58±6.70	6.23±3.26	6.87±1.35	30.08±3.36	51.23±5.25
	10	5.70±8.29	6.54±1.36	6.46±3.58	29.89±3.76	51.40±6.14

Table S4: Relaxation timescales (µs) for 5-macrostate BHMSM models.

K-means	HMM Lag (ns)	t1	t2	t3	t4
100	8	2.69±2.10	0.99±0.45	0.29±0.02	0.19±0.01
	10	2.74±2.11	0.86±0.48	0.29±0.03	0.20±0.01
500	8	2.46±2.38	0.87±0.34	0.25±0.02	0.17±0.01
	10	2.60±1.93	0.85±0.42	0.27±0.02	0.18±0.01
1000	8	2.55±2.29	0.90±0.39	0.24±0.02	0.16±0.01
	10	3.43±4.92	0.98±0.44	0.26±0.02	0.17±0.01

Table S5: State populations for 6-macrostate BHMSM models.

K-means	HMM Lag (ns)	S1	S2	S3	S4	S5	S6
100	8	2.84±0.86	5.22±0.88	3.47±4.52	3.91±2.37	23.35±3.00	61.21±5.37
	10	3.10±2.74	5.00±1.08	4.16±0.73	8.95±4.74	25.63±2.93	53.16±5.30
500	8	1.89±0.49	4.15±2.07	4.79±5.36	5.92±1.10	30.39±3.02	52.87±4.63
	10	1.80±0.60	5.73±1.50	12.29±16.14	3.15±1.85	28.07±5.69	48.96±9.65
1000	8	4.99±1.05	3.85±1.55	4.63±5.42	4.29±0.71	30.68±2.96	51.56±4.41
	10	1.70±1.33	3.35±1.88	5.52±1.59	9.59±14.73	28.54±5.46	51.31±9.15

Table S6: Relaxation timescales (µs) for 6-macrostate BHMSM models.

K-means	HMM Lag (ns)	t1	t2	t3	t4	t5
100	8	3.04±2.46	1.05±0.50	0.38±0.03	0.26±0.02	0.07±0.01
	10	2.39±1.01	0.62±0.32	0.32±0.04	0.22±0.02	0.06±0.00
500	8	2.20±2.10	0.85±0.30	0.25±0.02	0.17±0.01	0.06±0.01
	10	6.71±9.32	0.96±0.40	0.25±0.03	0.18±0.02	0.07±0.01
1000	8	2.01±2.18	0.64±0.17	0.21±0.02	0.16±0.02	0.05±0.01
	10	5.17±8.40	0.63±0.37	0.23±0.03	0.16±0.02	0.08±0.04

Table S7: State populations for 6-macrostate BHMSM models using different feature sets.

Feature Set	BHMSM lag (ns)	S1	S2	S3	S4	S5	S6
18 features	8	2.84 ± 0.86	5.22 ± 0.88	3.47 ± 4.52	3.91 ± 2.37	23.35 ± 3.00	61.21 ± 5.37
	10	3.10 ± 2.74	5.00 ± 1.08	4.16 ± 0.73	8.95 ± 4.74	25.63 ± 2.93	53.16 ± 5.30
94 features	8	0.42 ± 0.26	6.82 ± 4.42	6.02 ± 1.07	3.96 ± 1.47	4.70 ± 5.87	78.08 ± 7.52
	10	7.07 ± 7.15	6.54 ± 3.77	2.49 ± 0.58	4.19 ± 1.24	20.19 ± 2.96	59.53 ± 6.78
Backbone torsion	8	8.19 ± 10.13	6.53 ± 1.72	4.82 ± 4.50	8.34 ± 2.37	18.32 ± 6.44	53.80 ± 9.27
	10	19.54 ± 27.19	3.98 ± 4.03	19.56 ± 7.15	9.94 ± 4.21	14.30 ± 7.34	32.68 ± 11.72
Hydrophobic COM	8	5.66 ± 0.91	2.51 ± 1.33	3.63 ± 2.74	2.49 ± 3.54	5.42 ± 1.05	80.30 ± 5.12
	10	2.33 ± 3.59	2.77 ± 1.68	5.93 ± 4.70	6.12 ± 1.61	7.90 ± 12.11	74.96 ± 13.73

Table S8: Relaxation timescales (µs) for 6-macrostate BHMSM models constructed from different feature sets.

Feature Set	BHMSM lag (ns)	t1	t2	t3	t4	t5
18 features	8	3.04 ± 2.46	1.05 ± 0.50	0.38 ± 0.03	0.26 ± 0.02	0.07 ± 0.01
	10	2.39 ± 1.01	0.62 ± 0.32	0.32 ± 0.04	0.22 ± 0.02	0.06 ± 0.00
94 features	8	3.45 ± 2.50	1.16 ± 0.58	0.35 ± 0.05	0.09 ± 0.01	0.05 ± 0.01
	10	3.47 ± 2.31	0.79 ± 0.33	0.35 ± 0.05	0.11 ± 0.02	0.07 ± 0.01
Backbone torsion	8	2.83 ± 4.01	0.96 ± 0.48	0.49 ± 0.16	0.33 ± 0.04	0.28 ± 0.03
	10	64.12 ± 239.59	1.68 ± 0.74	0.84 ± 0.33	0.38 ± 0.06	0.25 ± 0.02
Hydrophobic COM	8	2.22 ± 2.20	0.88 ± 0.51	0.26 ± 0.03	0.10 ± 0.04	0.07 ± 0.01
	10	6.21 ± 8.06	1.73 ± 1.06	0.29 ± 0.05	0.19 ± 0.05	0.13 ± 0.04

Table S9: Stationary probabilities (π), free energies (G), lifetimes, average protein heavy-atom root-mean-square deviations (RMSDs) and radius of gyration (Rg) of the metastable states. The RMSDs were computed after alignment to the crystal structure.

State	π (%)	G (kcal/mol)	Lifetime (μs)	RMSD (nm)	Rg (nm)
S1	2.38 ± 0.78	1.946 ± 0.196	0.31 ± 0.03	0.429 ± 0.018	1.227 ± 0.011
S2	5.10 ± 0.90	1.492 ± 0.105	0.09 ± 0.01	0.401 ± 0.019	1.207 ± 0.013
S3	2.02 ± 2.14	2.045 ± 0.633	0.46 ± 0.15	0.430 ± 0.018	1.213 ± 0.011
S4	3.78 ± 2.38	1.670 ± 0.375	0.68 ± 0.13	0.453 ± 0.017	1.226 ± 0.010
S5	24.4 ± 2.97	0.558 ± 0.072	0.29 ± 0.03	0.394 ± 0.019	1.200 ± 0.011
S6	62.2 ± 4.83	0.000 ± 0.046	0.97 ± 0.12	0.395 ± 0.014	1.198 ± 0.010

Table S10: Druggability scores and cavity volumes computed using DoGSiteScorer for the top-ranked clusters across metastable states. Values are reported as mean and standard deviation calculated from the two highest-ranked clusters per state. The identified cavities closely correspond to the binding pocket described in the main text.

	**Cavity 1**		**Cavity 2**		**Cavity 3**	
**State**	Drug Score	V (Å^3)	Drug Score	V (Å^3)	Drug Score	V (Å^3)
S1	0.27 ± 0.00	99.97 ± 0.00	0.69 ± 0.00	112.64 ± 0.00	0.32 ± 0.02	153.54 ± 6.79
S2	0.71 ± 0.01	270.21 ± 100.56	–	–	0.50 ± 0.33	325.83 ± 268.72
S3	0.22 ± 0.00	214.21 ± 0.00	–	–	0.62 ± 0.18	341.12 ± 72.68
S4	0.35 ± 0.23	71.36 ± 43.63	0.61 ± 0.06	95.11 ± 2.09	0.34 ± 0.07	418.66 ± 92.91
S5	0.25 ± 0.06	144.61 ± 50.73	–	–	0.35 ± 0.13	425.25 ± 134.28
S6	0.36 ± 0.00	121.41 ± 0.00	–	–	0.57 ± 0.15	308.36 ± 71.41

Table S11: Cavity volumes, mean local hydrophobic densities, and cavity occupancies across metastable states. Values are reported as mean and standard error.

	**Cavity 1**			**Cavity 2**			**Cavity 3**		
**State**	V (Å^3)	Hyd. Density	Occ. (%)	V (Å^3)	Hyd. Density	Occ. (%)	V (Å^3)	Hyd. Density	Occ. (%)
S1	277.0 ± 7.0	28.78 ± 1.67	67.3	119.4 ± 3.5	35.26 ± 1.63	43.7	378.0 ± 9.4	49.43 ± 1.82	87.3
S2	267.5 ± 7.5	21.46 ± 1.39	66.7	49.8 ± 3.9	10.71 ± 2.07	12.0	418.6 ± 10.1	56.43 ± 2.18	91.7
S3	249.7 ± 7.7	22.82 ± 1.69	64.3	43.1 ± 4.4	7.95 ± 2.54	6.3	397.4 ± 9.9	56.62 ± 1.87	92.7
S4	300.9 ± 6.9	37.99 ± 1.65	73.3	136.6 ± 4.1	36.42 ± 1.39	45.3	385.5 ± 8.8	50.31 ± 1.58	93.0
S5	277.0 ± 7.1	20.81 ± 1.45	76.3	38.0 ± 3.6	4.09 ± 1.13	4.3	411.2 ± 10.1	57.07 ± 1.90	95.3
S6	255.5 ± 6.0	16.37 ± 1.19	72.7	40.6 ± 5.5	4.02 ± 0.99	4.7	419.6 ± 9.7	57.51 ± 1.98	97.3

Table S12: Mean First Passage Times (MFPT (μs)) between the metastable states. Values are reported as means and standard deviations.

**From / To**	S1	S2	S3	S4	S5	S6
S1	0.0 ± 0.0	0.81 ± 0.12	35.88 ± 8.70	27.60 ± 5.75	1.24 ± 0.16	1.44 ± 0.17
S2	17.65 ± 2.34	0.0 ± 0.0	41.66 ± 9.81	36.06 ± 6.73	0.56 ± 0.05	0.75 ± 0.06
S3	9.13 ± 1.98	1.63 ± 0.46	0.0 ± 0.0	12.79 ± 4.53	2.08 ± 0.46	2.30 ± 0.49
S4	6.75 ± 1.19	1.96 ± 0.35	17.35 ± 6.47	0.0 ± 0.0	2.37 ± 0.36	2.55 ± 0.37
S5	19.11 ± 2.39	1.65 ± 0.28	41.73 ± 9.09	37.63 ± 6.84	0.0 ± 0.0	0.61 ± 0.04
S6	19.86 ± 2.41	2.35 ± 0.34	41.59 ± 8.64	38.33 ± 6.86	1.07 ± 0.13	0.0 ± 0.0

Table S13: The slowest relaxation timescales of the 6-microstates model. Error represents standard deviations.

Timescale	Value (μs)
1	1.74 ± 0.36
2	0.40 ± 0.04
3	0.36 ± 0.02
4	0.27 ± 0.02
5	0.08 ± 0.01

Supplementary Figures

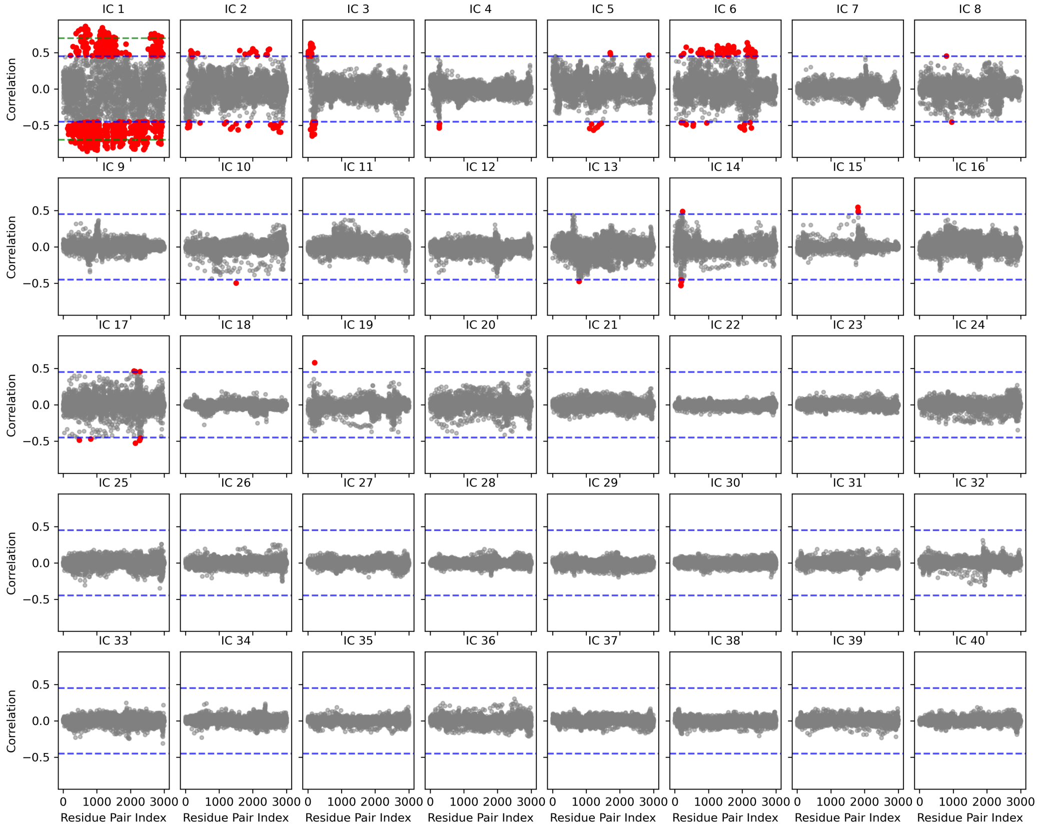

Figure S1: Feature–TICA correlations of residue pairs for the first 40 independent components. Correlation values above 0.45 are shown in red, and values below 0.45 are shown in gray. The horizontal blue line indicates the 0.45 threshold, and the green line indicates 0.70.

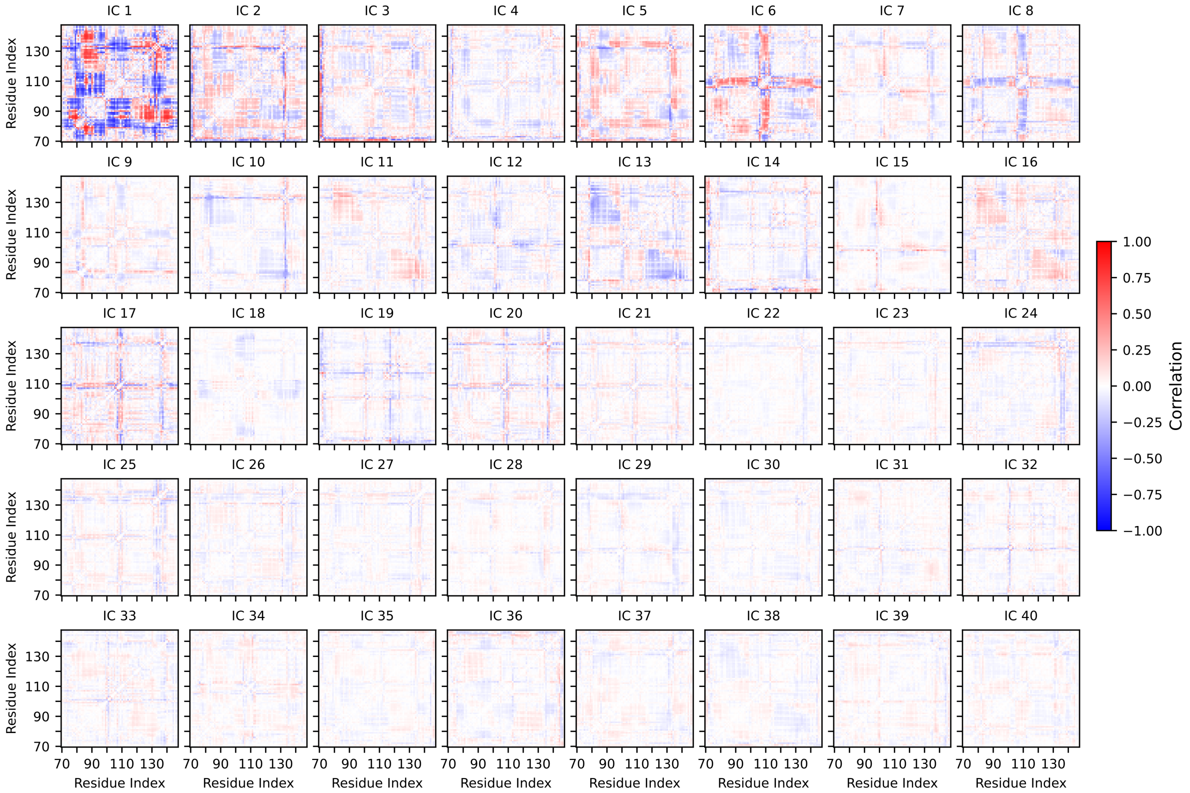

Figure S2: Feature-TICA correlations of residue pairs for the first 40 independent components, shown as a matrix. The color bar indicates the correlation values.

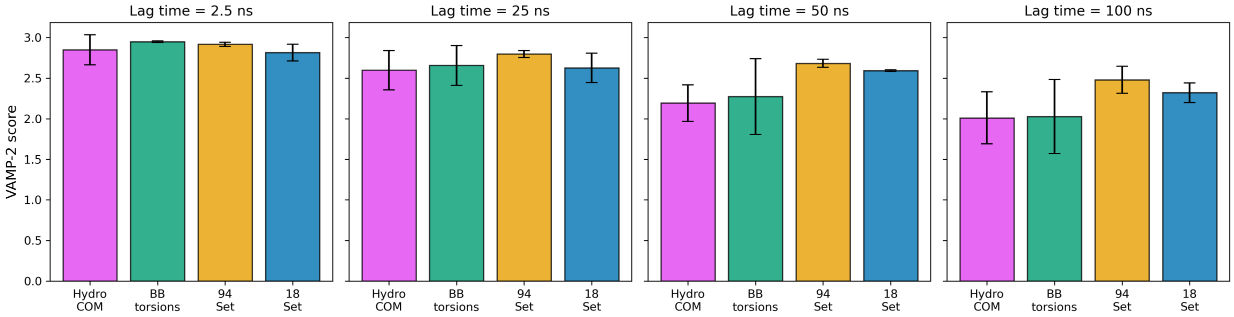

Figure S3: VAMP-2 scores for different feature sets. Bars represent the mean VAMP-2 score computed using a five-part cross-validation procedure. Error bars indicate the standard deviation across partitions. Feature sets include hydrophobic residue COM distances (Hydro. COM), backbone torsions (BB torsions), the 94-feature set, and the 18-feature set. VAMP-2 Scores were calculated in two dimensions.

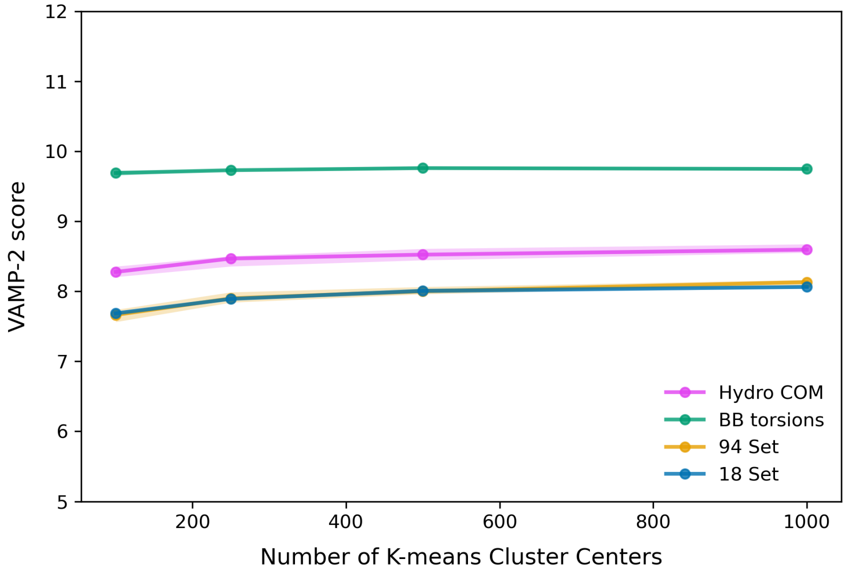

Figure S4: VAMP-2 scores as a function of the number of k-means clusters for different feature sets: hydrophobic residue COM distances (Hydro. COM), and backbone torsions (BB torsions), the 94-feature set, and the 18-feature set. Shaded regions represent 90% confidence intervals calculated from five independent k-means runs. VAMP-2 Scores are shown for a TICA lag time of 2.5 ns.

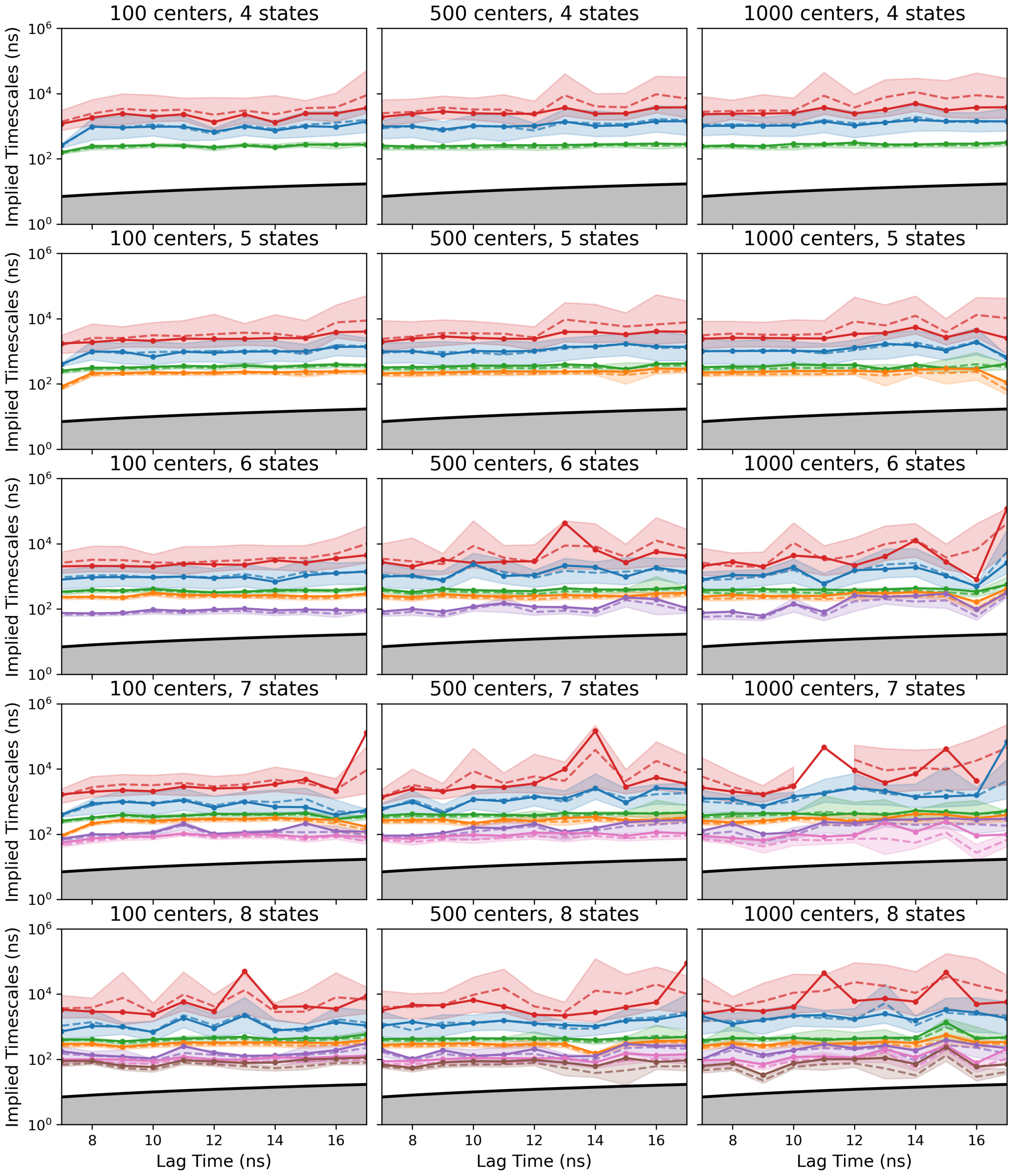

Figure S5: Implied timescales of the slowest dynamical processes for BHMSM with 6 states for the 18-feature set. The ITS were calculated with a TICA lag time of 2.5 ns and 100, 500, 1000 microstates. Dashed lines represent BHMSM sample means, while solid lines correspond to maximum-likelihood estimates. Shaded areas indicate 90% confidence intervals. The black line with the grey-shaded region denotes processes faster than the lag time.

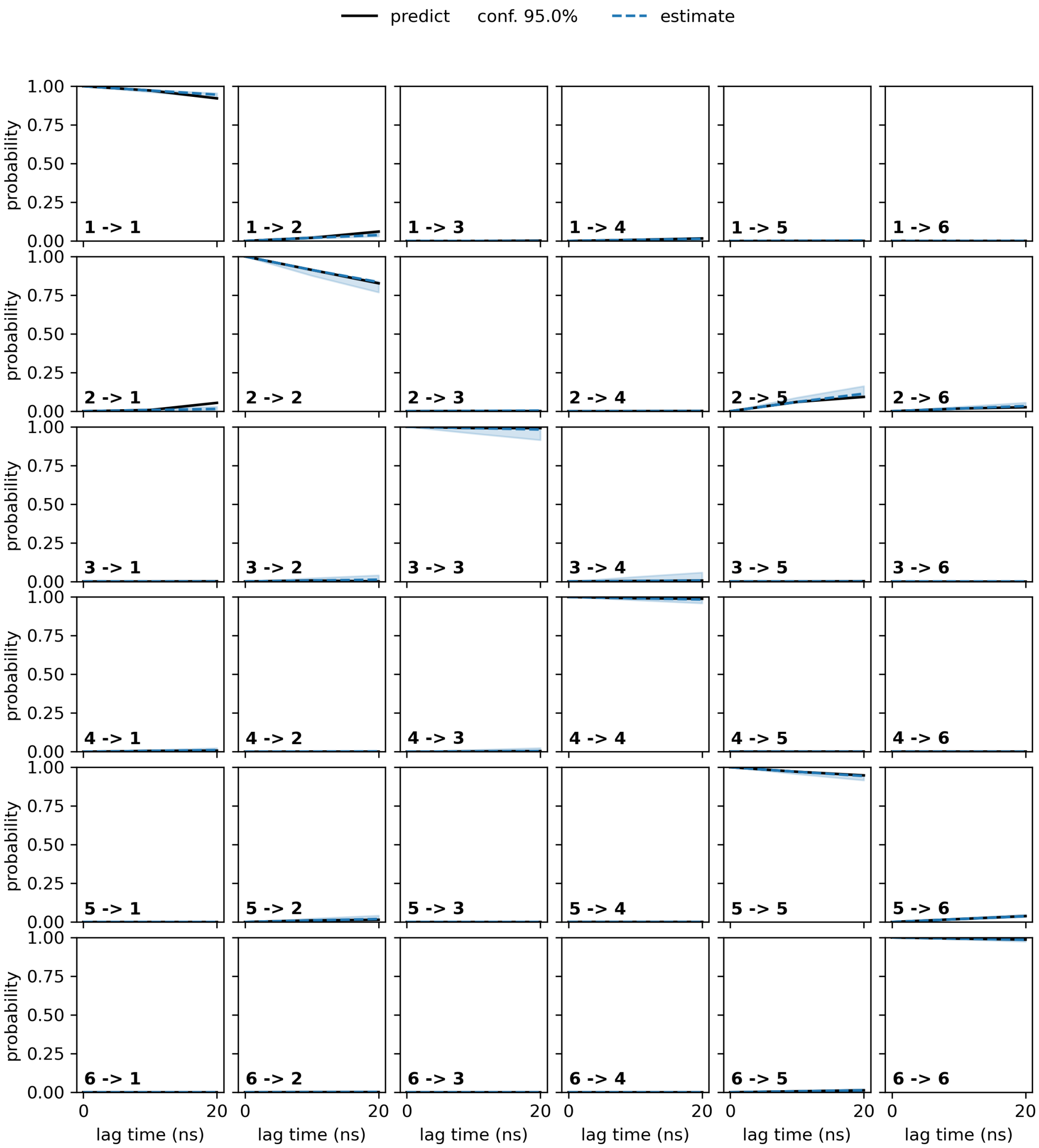

Figure S6: Chapman-Kolmogorov test for Bayesian Hidden Markov state model (BHMSM) validation at a lag time of 10 ns with six states. The estimated models are represented by blue solid lines, while the predictions are shown as black dashed lines. The shaded regions represent the 95% confidence intervals of the estimated models.

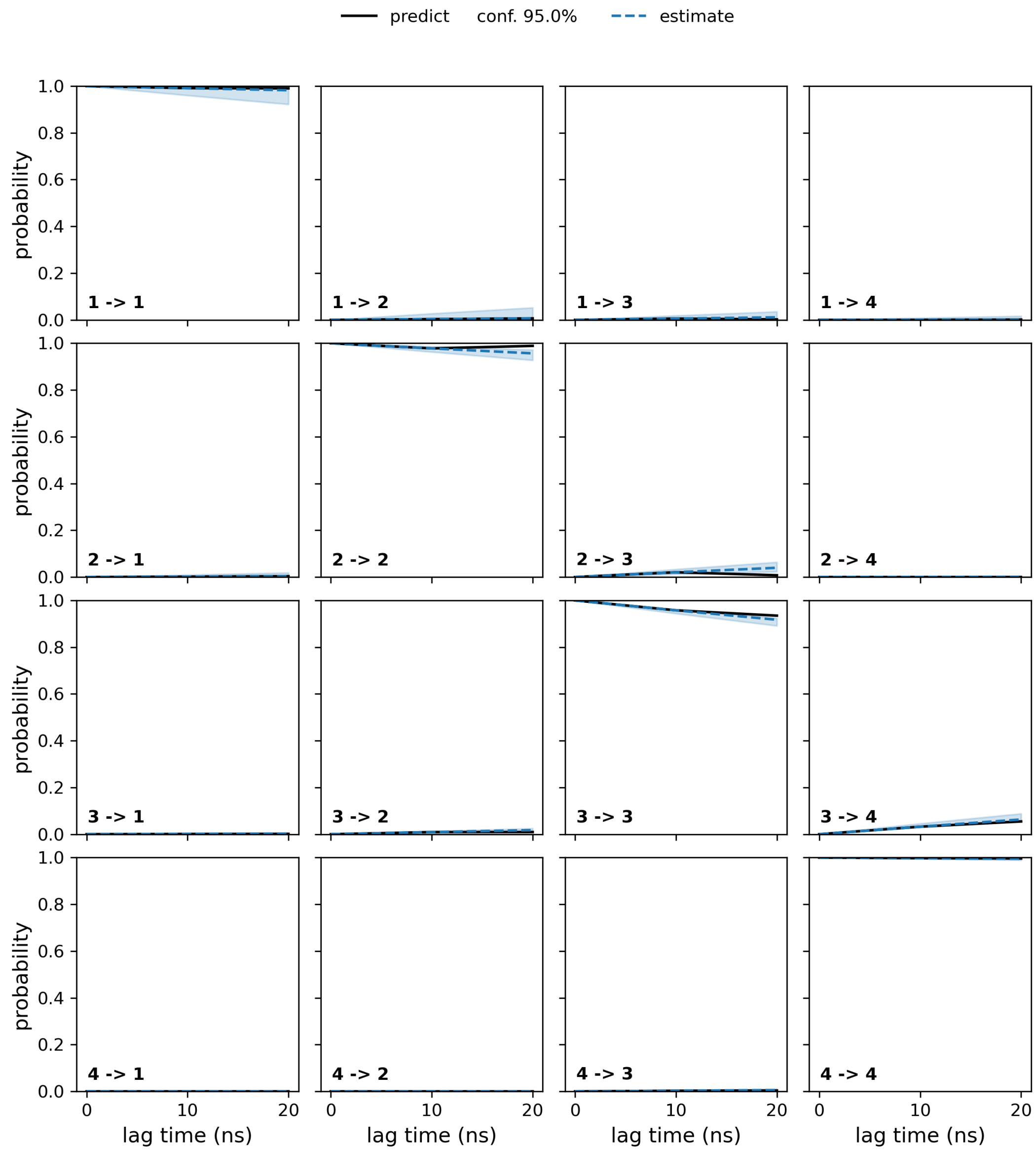

Figure S7: Chapman-Kolmogorov test for BHMSM validation at a lag time of 10 ns with four states. The estimated models are represented by blue solid lines, while the predictions are shown as black dashed lines. The shaded regions represent the 95% confidence intervals of the estimated models.

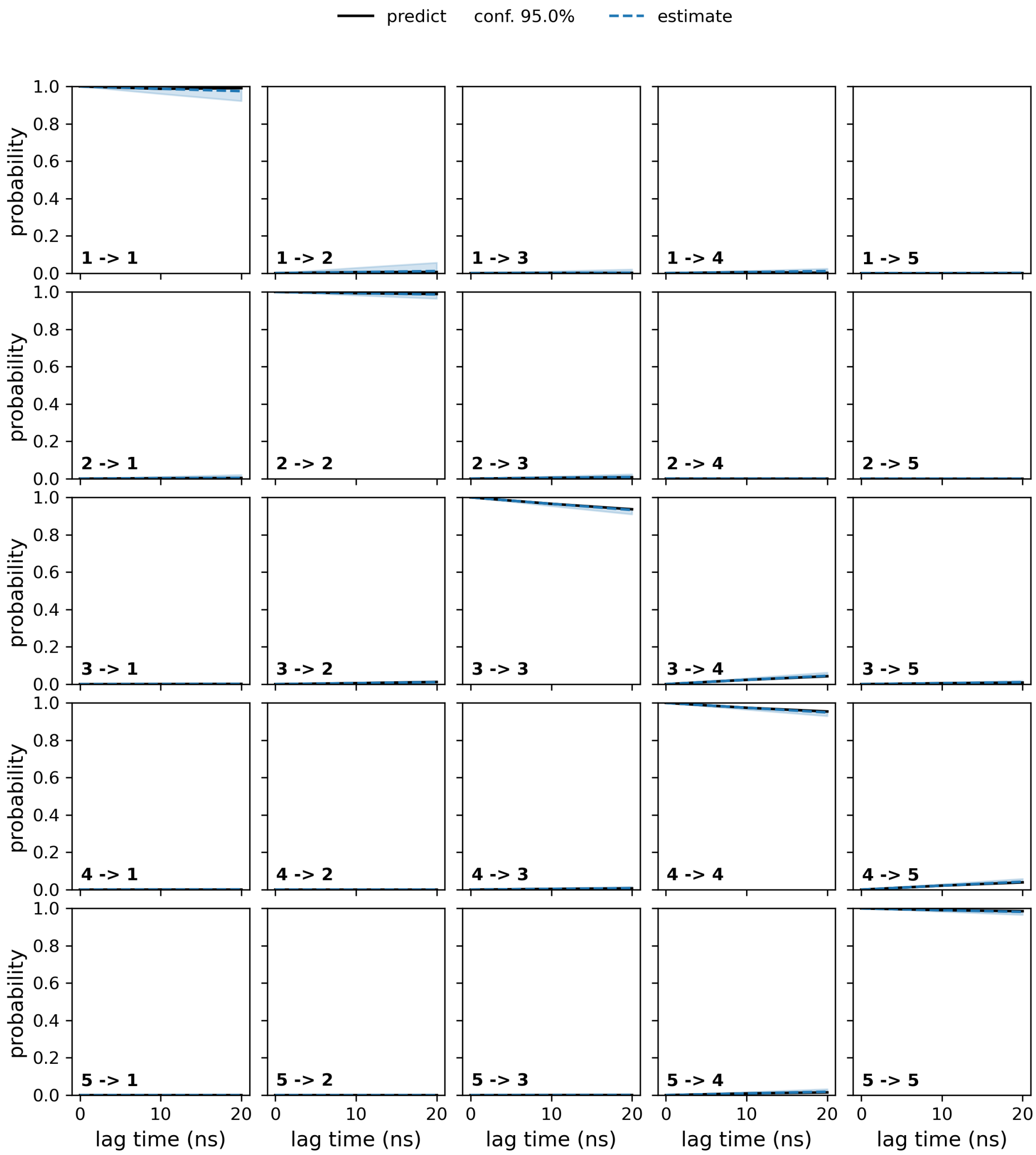

Figure S8: Chapman-Kolmogorov test for BHMSM validation at a lag time of 10 ns with five states. The estimated models are represented by blue solid lines, while the predictions are shown as black dashed lines. The shaded regions represent the 95% confidence intervals of the estimated models.

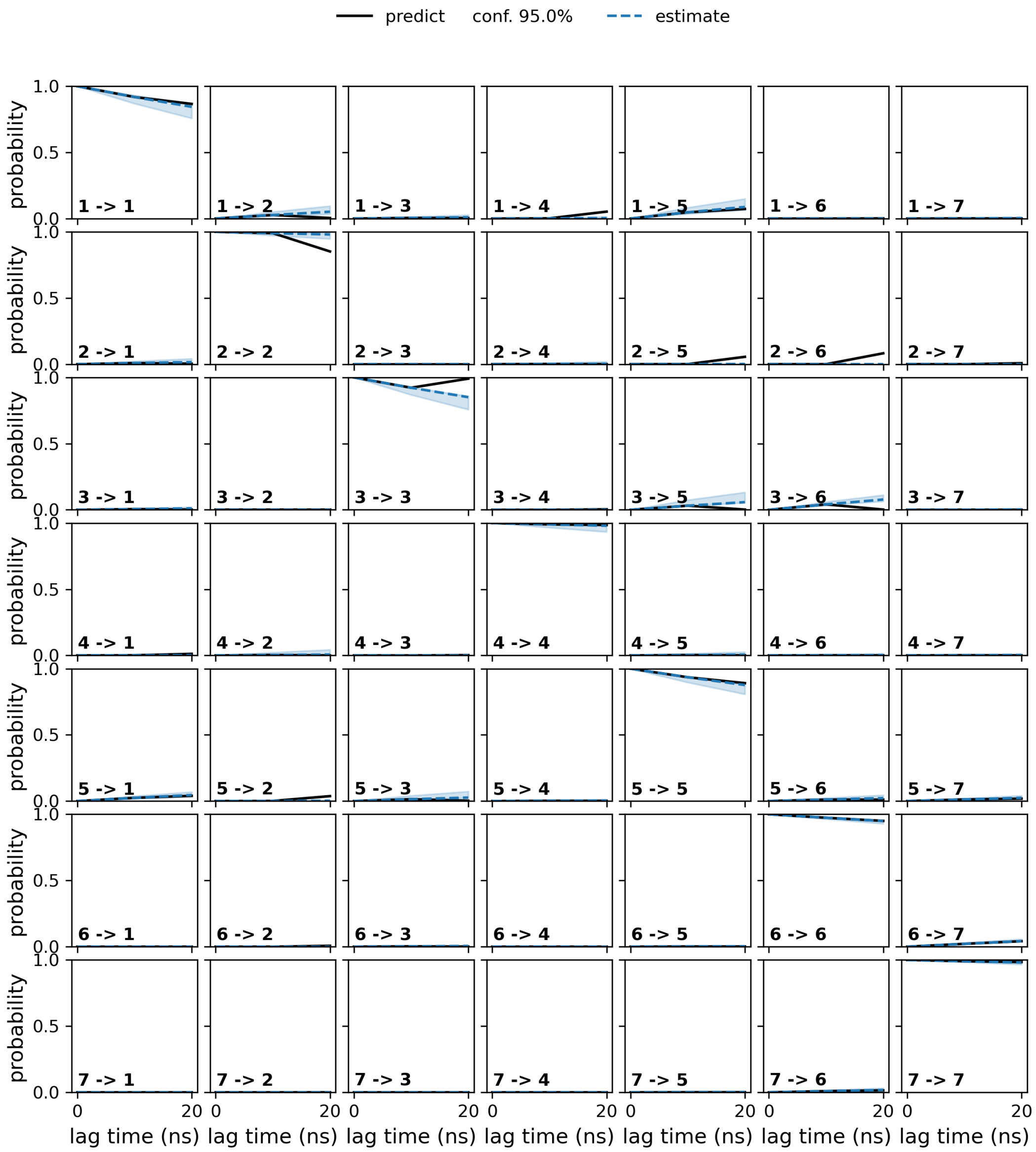

Figure S9: Chapman-Kolmogorov test for BHMSM validation at a lag time of 10 ns with seven states. The estimated models are represented by blue solid lines, while the predictions are shown as black dashed lines. The shaded regions represent the 95% confidence intervals of the estimated models.

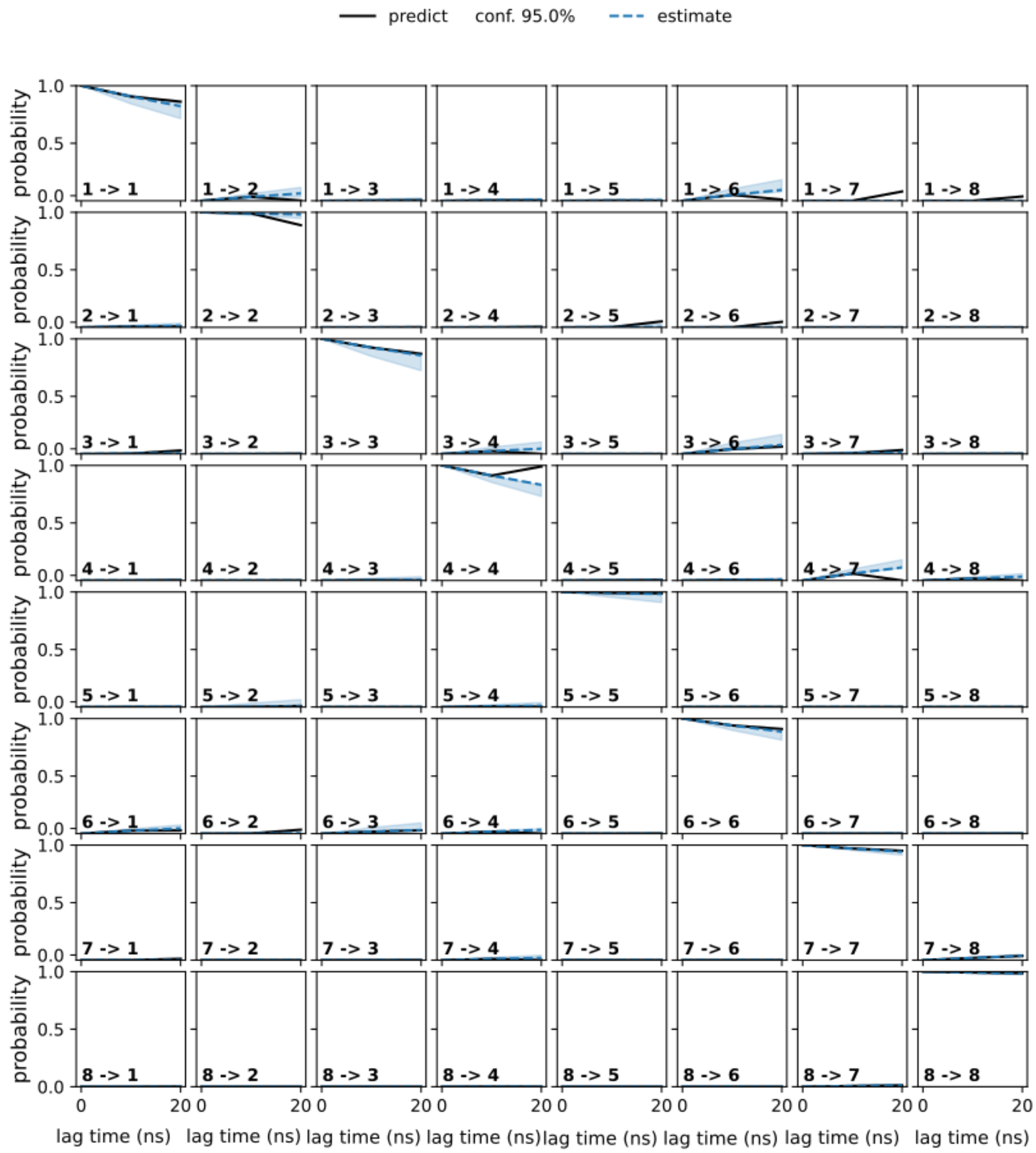

Figure S10: Chapman-Kolmogorov test for BHMSM validation at a lag time of 10 ns with eight states. The estimated models are represented by blue solid lines, while the predictions are shown as black dashed lines. The shaded regions represent the 95% confidence intervals of the estimated models.

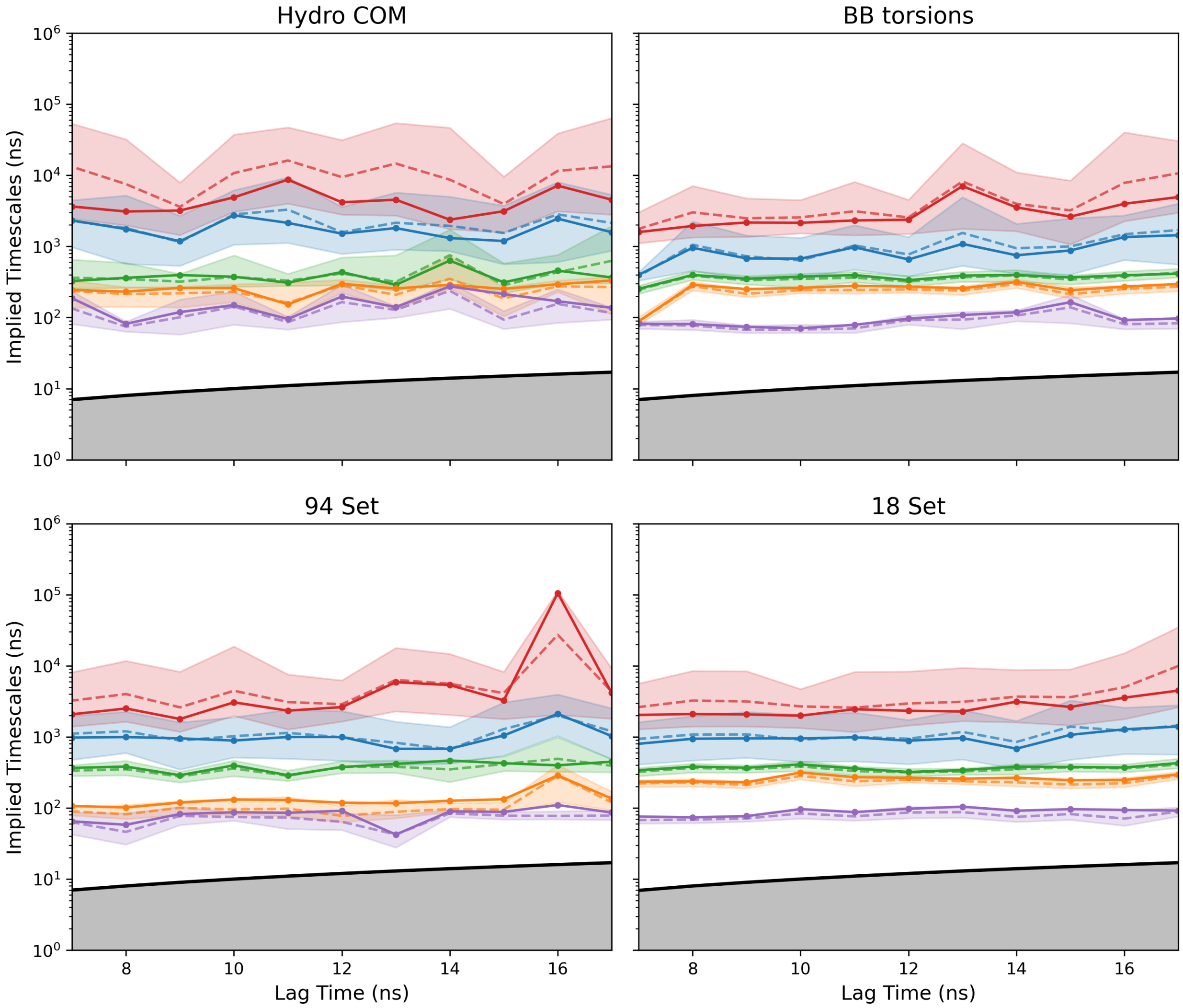

Figure S11: Implied timescales of the slowest dynamical processes for BHMSM with 6 states for feature sets: hydrophobic residue COM distances (Hydro. COM), and backbone torsions (BB torsions), the 94-feature set, and the 18-feature set. The ITS were calculated with a TICA lag time of 2.5 ns and 100 microstates. Dashed lines represent BHMSM sample means, while solid lines correspond to maximum-likelihood estimates. Shaded areas indicate 90% confidence intervals. The black line with the grey-shaded region denotes processes faster than the lag time.

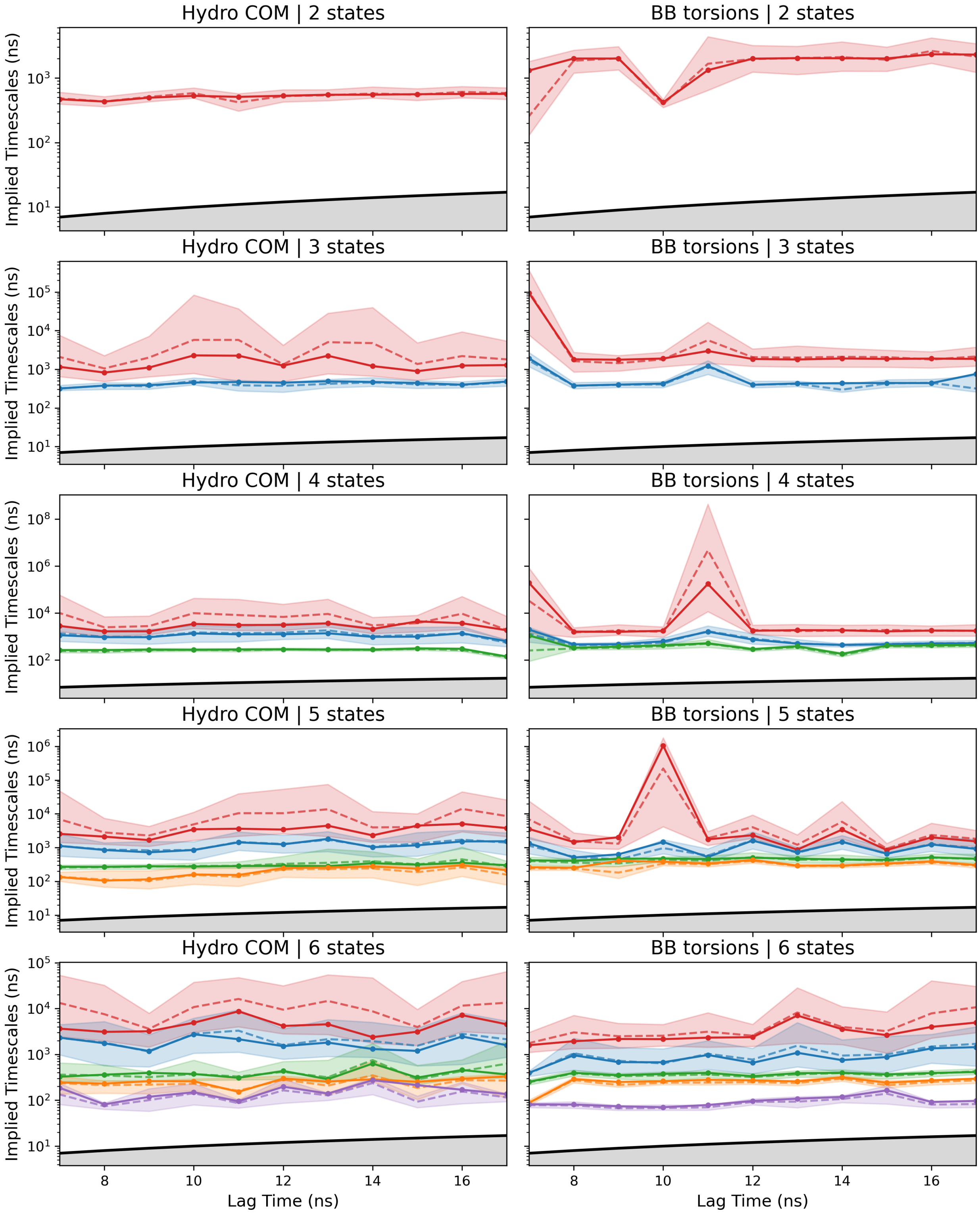

Figure S12: Implied timescales of the slowest dynamical processes for BHMSM with 2, 3, 4, 5, and 6 states for feature sets: hydrophobic residue COM distances (Hydro COM), and backbone torsions (BB torsions). The ITS were calculated with a TICA lag time of 2.5 ns and 100 microstates. Dashed lines represent BHMSM sample means, while solid lines correspond to maximum-likelihood estimates. Shaded areas indicate 90% confidence intervals. The black line with the grey-shaded region denotes processes faster than the lag time.

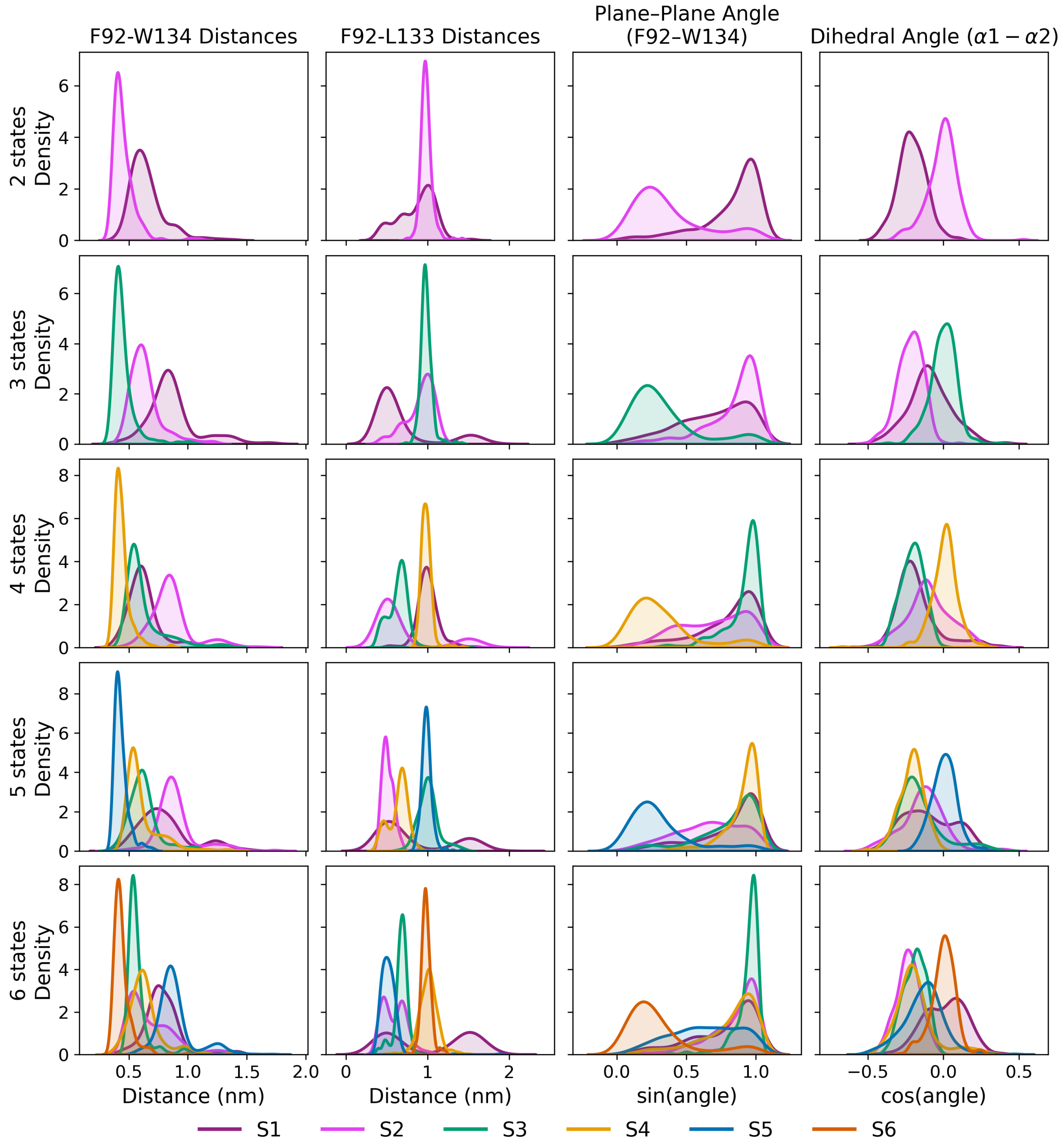

Figure S13: Distributions of structural features for the hydrophobic residue COM distances feature set across models with 2, 3, 4, 5, and 6 metastable states: The side-chain COM distances between F92-W134, the side-chain COM distances between F92-W133, the angle between the planes of W92 and W134 and the dihedral angle defined by the Cα atoms of F86-F96 and A119-L129, describing helix motion. The color code used for the six metastable states is consistent across panels: S1 (sky blue), S2 (pink), S3 (green), S4 (orange), S5 (blue), and S6 (red).

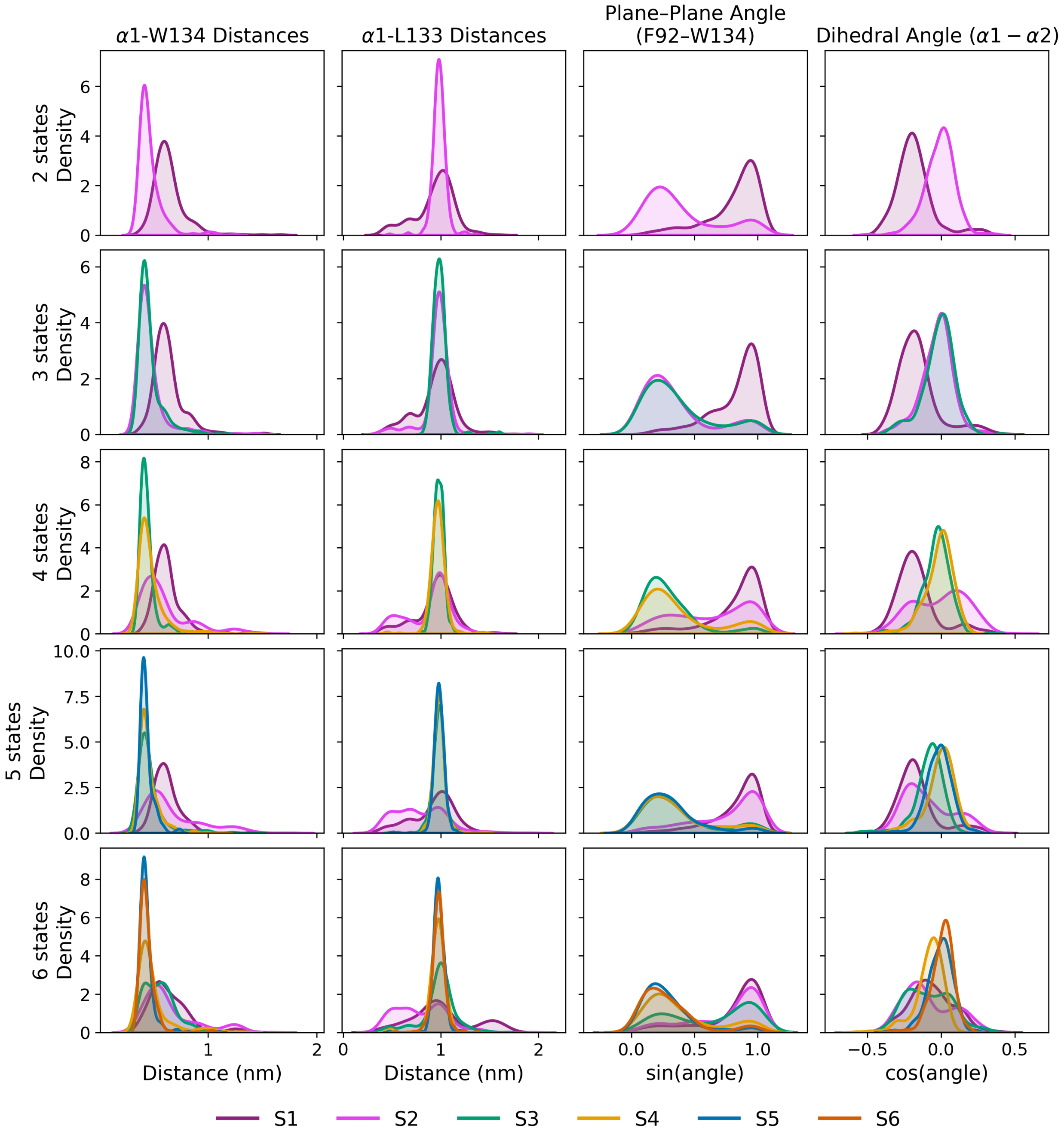

Figure S14: Distributions of structural features for the backbone torsions feature set across models with 2, 3, 4, 5, and 6 metastable states: The side-chain COM distances between F92-W134, the side-chain COM distances between F92-W133, the angle between the planes of W92 and W134 and the dihedral angle defined by the Cα atoms of F86-F96 and A119-L129, describing helix motion. The color code used for the six metastable states is consistent across panels: S1 (sky blue), S2 (pink), S3 (green), S4 (orange), S5 (blue), and S6 (red).

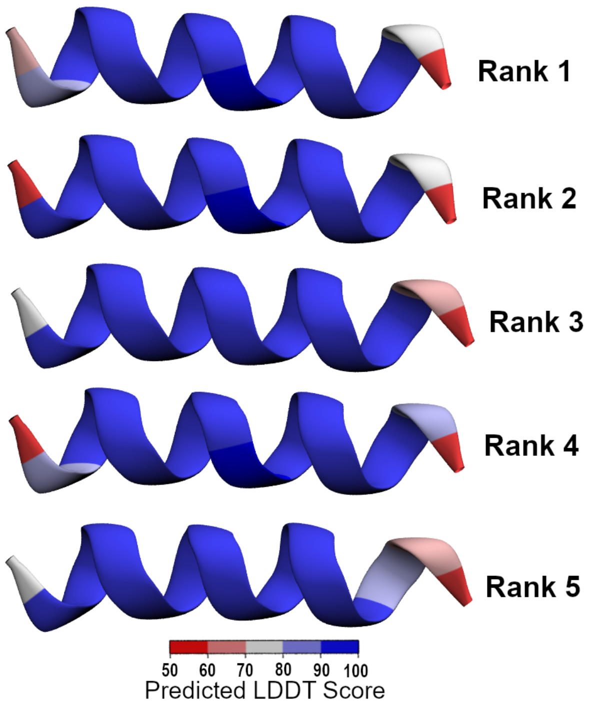

Figure S15: Predicted AlphaFold2 structures for residues 119-134, encompassing the α2-helix and loop5. Structures are colored according to their predicted LDDT (local distance difference test) scores (red to blue), where scores > 90 indicate high confidence, and scores between 70-90 indicate regions expected to be modeled accurately.

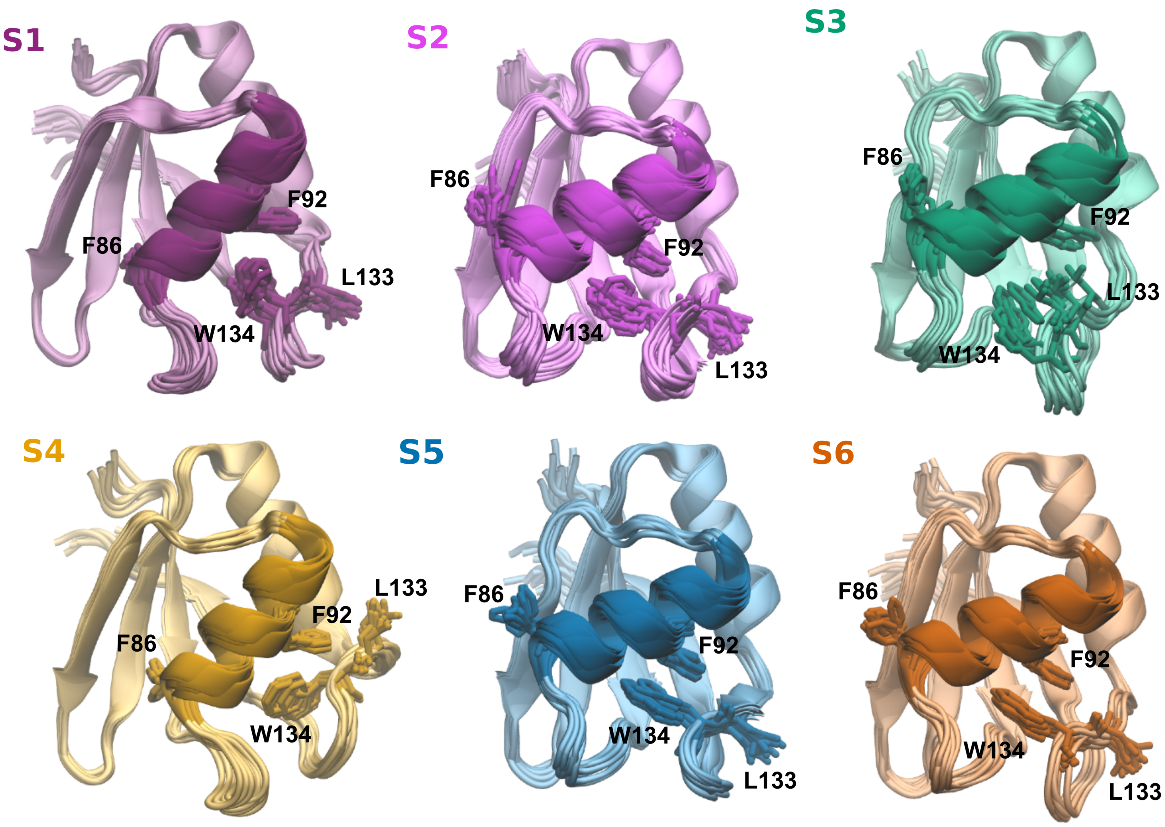

Figure S16: Representative conformations of each metastable state are shown as cartoons, with residues F86, F92, L133, and W134 highlighted as sticks. α1-helix is also highlighted in the cartoon depiction.

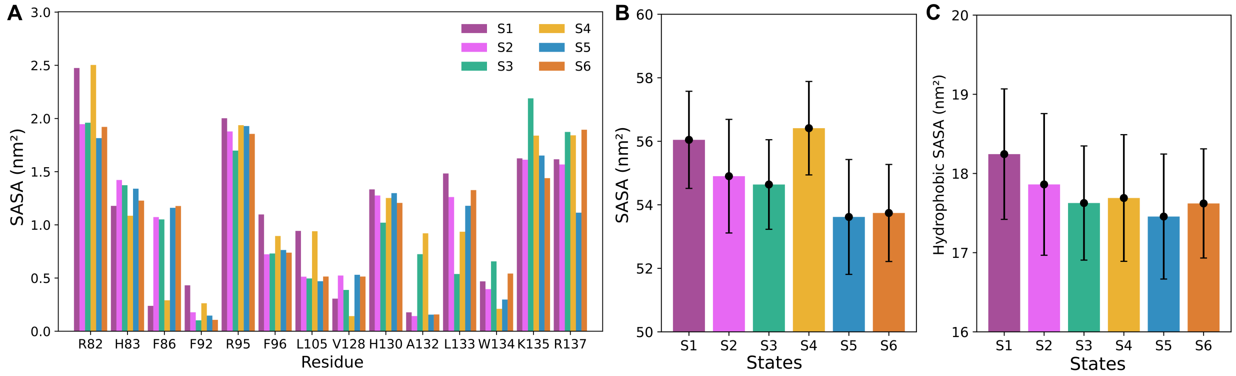

Figure S17: (A) Average solvent-accessible surface area (SASA) of residues showing significant differences (> 0.3 nm^2) across the metastable states. (B) Average total SASA of the protein in each metastable state. (C) Average SASA of the protein hydrophobic residues in each metastable state. Error bars represent standard deviations.

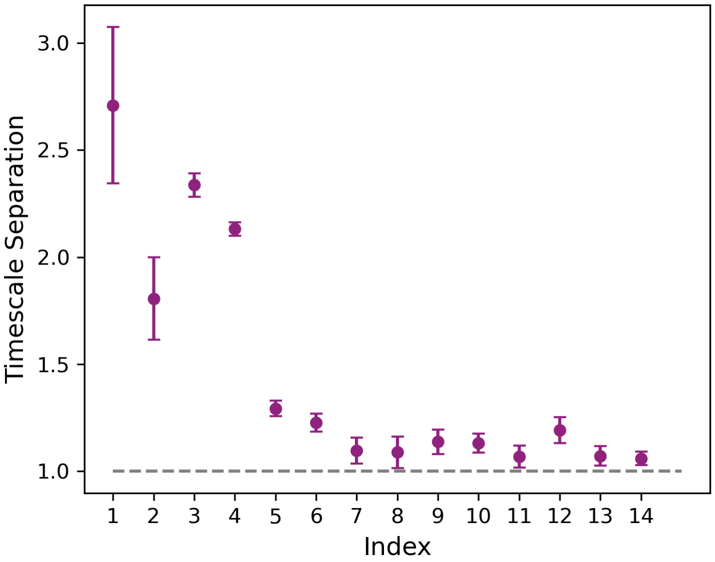

Figure S18: The timescales spectral gap between the successive implied timescales of the Markov state model. Error bars represent standard deviations.
